# Cascaded, Feedback-Driven, and Spatially Localized Emergence of Constitutional Dynamic Networks Driven by Enzyme-Free Catalytic DNA Circuits

**DOI:** 10.1021/jacs.3c02083

**Published:** 2023-05-31

**Authors:** Zhixin Zhou, Nina Lin, Yu Ouyang, Songqin Liu, Yuanjian Zhang, Itamar Willner

**Affiliations:** † School of Chemistry and Chemical Engineering, Southeast University, Nanjing 211189, China; ‡ Institute of Chemistry, The Hebrew University of Jerusalem, Jerusalem 91904, Israel

## Abstract

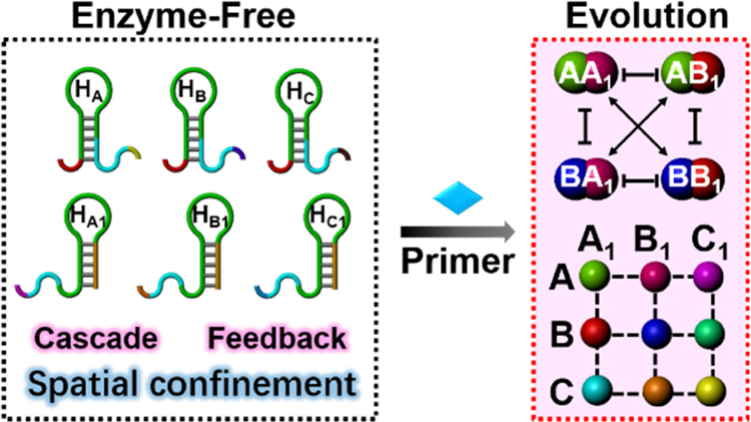

The enzyme-free catalytic hairpin assembly (CHA) process is introduced as a functional reaction module for guided, high-throughput, emergence, and evolution of constitutional dynamic networks, CDNs, from a set of nucleic acids. The process is applied to assemble networks of variable complexities, functionalities, and spatial confinement, and the systems provide possible mechanistic pathways for the evolution of dynamic networks under prebiotic conditions. Subjecting a set of four or six structurally engineered hairpins to a promoter P_1_ leads to the CHA-guided emergence of a [2 × 2] CDN or the evolution of a [3 × 3] CDN, respectively. Reacting of a set of branched three-arm DNA-hairpin-functionalized junctions to the promoter strand activates the CHA-induced emergence of a three-dimensional (3D) CDN framework emulating native gene regulatory networks. In addition, activation of a two-layer CHA cascade circuit or a cross-catalytic CHA circuit and cascaded driving feedback-driven evolution of CDNs are demonstrated. Also, subjecting a four-hairpin-modified DNA tetrahedron nanostructure to an auxiliary promoter strand simulates the evolution of a dynamically equilibrated DNA tetrahedron-based CDN that undergoes secondary fueled dynamic reconfiguration. Finally, the effective permeation of DNA tetrahedron structures into cells is utilized to integrate the four-hairpin-functionalized tetrahedron reaction module into cells. The spatially localized miRNA-triggered CHA evolution and reconfiguration of CDNs allowed the logic-gated imaging of intracellular RNAs. Beyond the bioanalytical applications of the systems, the study introduces possible mechanistic pathways for the evolution of functional networks under prebiotic conditions.

## Introduction

Dynamic networks driven by intercommunicating nucleic acid–protein or protein–protein complexes play roles in biological transformations such as gene expression^1−3^ or cell apoptosis.^4,5^ Complex chemical principles guide these dynamic networks. These include adaptive^6,7^ features, spatial confinement,^8^ oscillatory,^9^ feedback,^10^ intercommunication between networks,^11^ and the out-of-equilibrium dissipative operation of dynamic circuits.^12,13^ Substantial research efforts are directed to the development of artificial biomimetic dynamic networks emulating functions of the native systems,^14^ as a part of the rapidly advancing field of System Chemistry.^15−17^

**Scheme 1:**
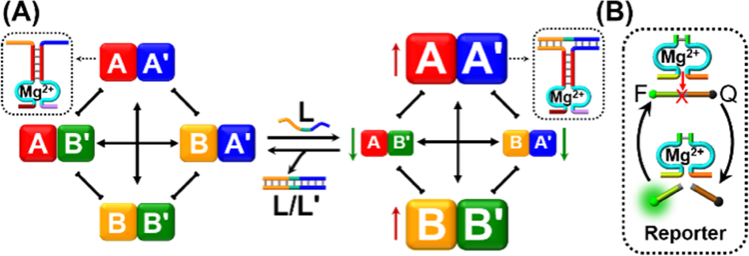
(A) Reversible Reconfiguration of a Nucleic Acid-Based Constitutional Dynamic Network (CDN) Using Fuel/Antifuel Strands as Triggers. (B) Probing the Compositions of CDNs by Following the Activities of Mg^2+^-Ion-Dependent DNAzyme Units Linked as Reporter Units to the Constituents

The information encoded in the base sequences of nucleic acids provides substantial structural and functional information into the biopolymer. These include dictated base-paired duplex formation and strand displacement,^18−20^ stimuli-triggered reconfiguration of nucleic acid strands into supramolecular structures such as ion-triggered formation of G-quadruplexes,^21^ pH-stimulated formation of i-motif structures,^22,23^ or pH/strand-induced formation and dissociation of C–G·C or T–A·T triplexes.^24^ Also, intercalation of photoisomerizable units, such as *trans/cis*-azobenzene compounds, was applied to switch duplex/single-stranded transitions.^25,26^ Within the efforts to emulate native networks by synthetic means, nucleic acid-based constitutional dynamic networks (CDNs) find growing interest.^14^ The simplest [2 × 2] DNA-based CDN comprises four components A, A′, B, and B′ that are structurally engineered to form four dynamically intercommunicated and equilibrated constituents AA′, AB′, BA′, and BB′, Scheme 1A. By the integration into one of the constituents, e.g., AA′, a responsive site that is stabilized or destabilized by an auxiliary pair of switchable trigger, L or L′, the equilibrated CDN is reversibly dynamically reconfigured to an adaptive framework responding to the auxiliary trigger.^27^ By engineering a cofactor-dependent DNAzyme unit into each of the constituent structures, a versatile reporter unit transducing the dynamic, adaptive, concentration changes of the constituents was demonstrated (by following the rates of cleavage of fluorophore/quencher-modified substrates and applying appropriate calibration curves, Scheme 1B). CDNs of enhanced complexities such as [3 × 3] CDNs^28^ were reported, and the dynamic networks were reconfigured by diverse triggers, such as fuel-strands, G-quadruplexes, or light.^27,29^ The synthetic CDN frameworks demonstrated adaptive,^27^ hierarchically adaptive,^28^ and intercommunicating mechanisms,^30^ mimicking native networks. In addition, different applications of CDNs were demonstrated, including network-guided biocatalytic cascades^31^ and dynamic DNA-based hydrogel matrices exhibiting switchable stiffness properties for self-healing and controlled drug-release.^32^

Key issues associated with the development of dynamic networks rest, however, on the mechanistic emergence of networks and particularly the functional integration of the dynamic frameworks into cells or cell-like containments (protocells) as models for primordial life. Indeed, several studies reported on the application of DNA polymerase/nicking machineries and a nucleic acid “pool” as a source for the guided emergence of CDNs,^33,34^ and the integration of polymerization machineries into protocells was demonstrated.^35^ Nevertheless, since enzymes were absent under prebiotic evolution, the search for nonenzymatic pathways for the emergence of networks, and particularly functional dynamic networks, needs to be demonstrated. Specifically, the discovery of catalytic properties of RNA^36^ shaped the concept “RNA world”,^37,38^ where the interaction between RNA and prebiotically synthesized amino acid provided supramolecular protoribosome structures^39^ for the evolution of peptides/proteins. Furthermore, evolution of life would require the integration of the protoribosome systems into cell-like containments where enhanced chemical reactivities proceed through the concentration of chemical ingredients in confined environments.^40^ The programmability of nucleic acid sequences and the understanding of the thermodynamic and kinetic properties embedded in the nucleic acids provide a means to rationally design molecular reaction pathways.^41^ Several nonenzymatic isothermal catalytic DNA circuits were developed, including the hybridization chain reaction (HCR),^42,43^ the catalytic hairpin assembly (CHA),^44,45^ and the entropy-driven catalysis.^18,46^ The CHA reaction involves two appropriately designed hairpins that are kinetically trapped in the absence of a DNA catalyst. In the presence of an auxiliary DNA as the catalyst, the opening and cross-hybridization of the hairpins proceed while recycling the catalyst strand. The CHA concept has been extended to include more than two hairpins in the oligomerization process^47^ and the intercommunication of the CHA-activated layers.^48^ Diverse applications of the CHA process were reported, including the operation of a DNA walker,^49^ tailoring amplified sensing platforms,^50,51^ intracellular imaging,^47,48,52−54^ and activation of a logic circuit.^55^

Here, we wish to report on the use of the CHA process as an effective nonenzymatic catalytic tool for the high-throughput emergence and evolution of CDNs. We demonstrate the use of primers and the CHA process for the emergence of complex [3 × 3] CDNs (comprising nine subnetworks) and the primer-guided emergence of three-arm junction-based 3D CDN that mimics gene regulatory networks. In addition, the cascaded and feedback-driven emergence of CDN was rationally designed using a self-catalytic two-layer CHA cascade circuit and a cross-catalytic CHA circuit. Besides the diversity of possible CDN structures, a spatially confined DNA tetrahedron architecture for the fast emergence of CDN was designed by organizing reactive DNA hairpins on a tetrahedron nanostructure. By coupling enzyme-free signal amplification and hierarchical adaptive control over the emergence and composition of CDNs, a localized CHA was developed for the logic-gated intracellular imaging of miRNAs. The study not only introduces concepts for the possible evolution of adaptive dynamically equilibrated CDNs under prebiotic conditions, in a world of nucleic acids, but also adds tools to integrate reaction moduli into cells or cell-like containments for the self-evolution and emergence of functional networks for biomedical applications.

## Results and Discussion

**Figure 1:**
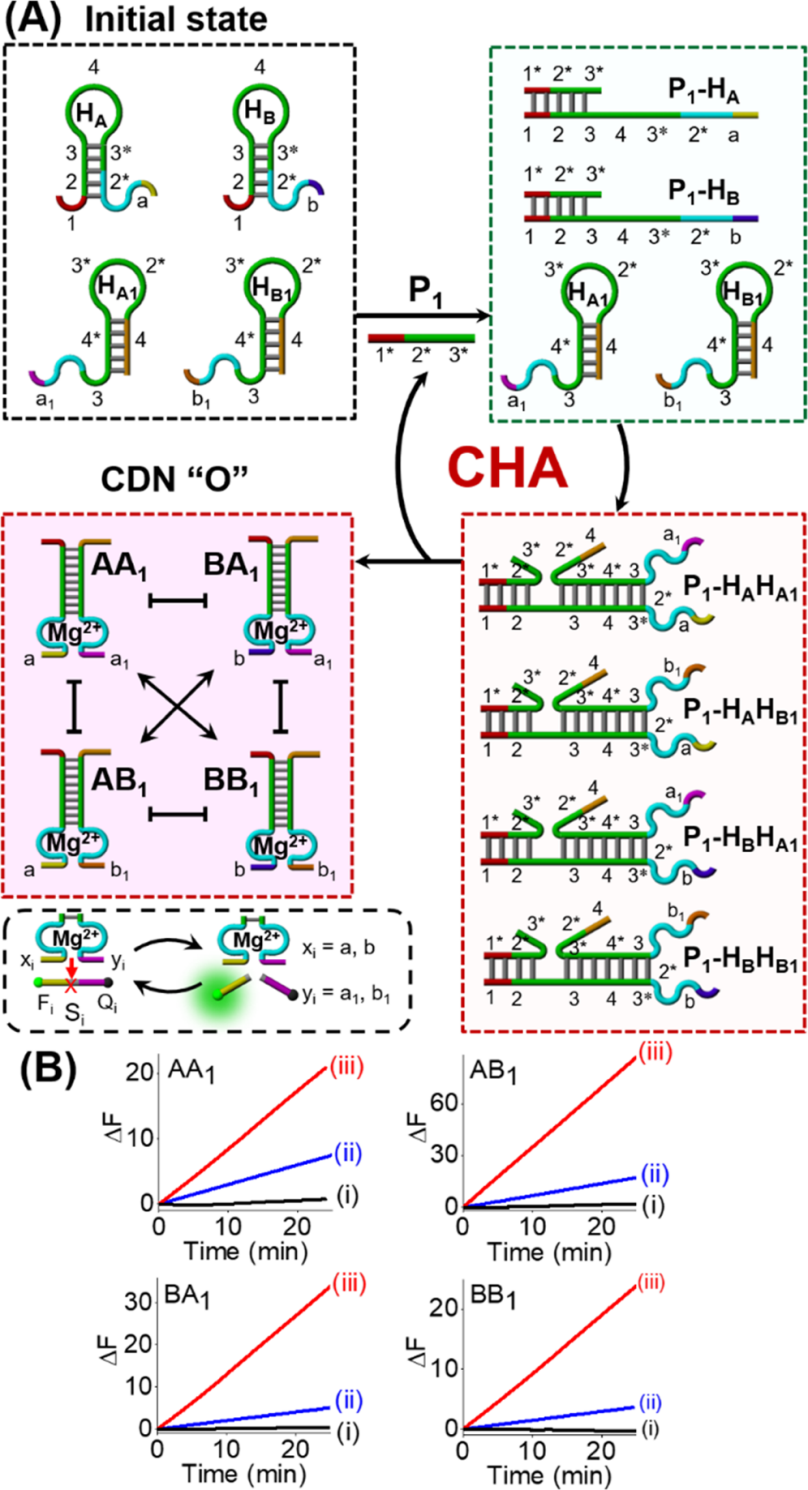
(A) Schematic of amplified primer-P_1_-guided evolution of a [2 × 2] CDN “O” using the enzyme-free CHA reaction and a set of four hairpin structures. (B) Time-dependent fluorescence changes generated by DNAzyme reporter units associated with four constituents of the CDN “O” evolved by enzyme-free CHA: (i) in the absence of P_1_, (ii) in the presence of 0.01 μM P_1_, and (iii) in the presence of 1 μM P_1_.

Figure 1A depicts the simplest system for the enzyme-free catalyzed emergence of a [2 × 2] CDN “O” using the CHA reaction. The parent reaction module consists of a set of four appropriately designed hairpin motifs, H_A_, H_A1_, H_B_, and H_B1_, which include the Mg^2+^-ion-dependent DNAzyme subunits in a caged, inactive configuration. The primer P_1_ opens the hairpins H_A_ and H_B_ to form the duplexes P_1_–H_A_ and P_1_–H_B_ acting as assembly initiators for H_A1_ and H_B1_, thus generating four transient intermediate three-component complexes P_1_–H_A_H_A1_, P_1_–H_A_H_B1_, P_1_–H_B_H_A1_, and P_1_–H_B_H_B1_. Meanwhile, the autonomous opening of four hairpin motifs disassociates the primer P_1_ from complexes to catalyze the next assembly circle, resulting in the continuous, amplified high-throughput assembly of four duplex constituents AA_1_, AB_1_, BA_1_, and BB_1_, comprising the dynamically equilibrated CDN “O”. Each of these constituents includes a different DNAzyme unit that serves as a “reporter” unit for the quantitative evaluation of the concentrations of the respective constituents in the CDN “O”. The DNAzyme reporter units differ in the arms, *x_i_* and *y_i_*, for the selective binding of the fluorophore/quencher (*F_i_*/*Q_i_*)-functionalized substrates, *S_i_*. By following the rates of cleavage of the *F_i_*/*Q_i_*-functionalized substrates and using appropriate calibration curves correlating the cleavage rates of the respective substrates to the concentrations of the intact constituents, Figures S1 and S2, the concentrations of the constituents in the dynamically equilibrated CDN “O” were assessed. (For a detailed explanation to evaluate the concentrations of the constituents in the CDNs, see Page S7, Supporting Information.) Figure 1B shows the time-dependent fluorescence changes generated by the four DNAzyme reporter units associated with the equilibrated constituents of CDN “O” generated by the CHA reaction at variable concentrations of P_1_ shown in Figure 1A. By using the appropriate calibration curves in Figures S1 and S2, the concentrations of the constituents in the equilibrated CDN “O” were evaluated, and their concentrations are summarized in Table 1. Evidently, in the presence of 0.01 μM primer P_1_, the enzyme-free amplified CHA reaction leads to the evolved CDN “O” composed of the components A, A_1_, B, and B_1_ in the concentration range of ca 0.25 μM. That is, the amplified CHA process led to an ca 25-fold increase in the contents of the equilibrated CDN. Further support for the emergence of the CDN “O” was obtained from quantitative gel electrophoretic experiments, Figure S3, and the corresponding results are summarized in Table S1. Very good agreement between the contents of the constituents determined by the DNAzyme reporter units and by the quantitative electrophoretic experiments is demonstrated.

**Table 1:** Concentrations of the Constituents in CDN “O” Evolved by the P_1_-Triggered Activation of the CHA Reaction Shown in Figure 1A

P_1_	CDN	AA_1_	AB_1_	BA_1_	BB_1_
1.00	O^a^	0.46	0.58	0.57	0.48
0.01	O^a^	0.17	0.13	0.10	0.10

a The concentrations of the constituents (μM) were determined by the time-dependent fluorescence changes generated by the DNAzyme reporter units and using appropriate calibration curves in Figures S1 and S2.

**Figure 2:**
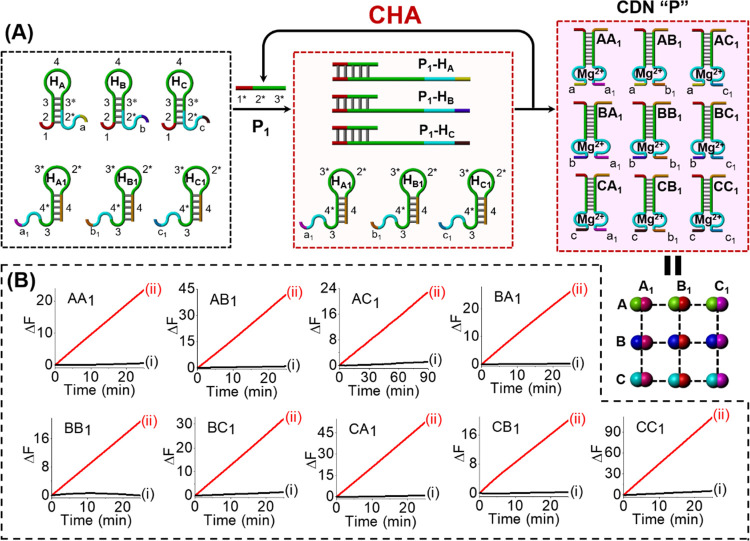
(A) Schematic primer-P_1_-guided emergence of a [3 × 3] CDN “P” using a set of six functional hairpin structures and the enzyme-free CHA reaction. (B) Time-dependent fluorescence changes generated by DNAzyme reporter units: (i) in the absence of primer P_1_, and (ii) in the presence of primer-P_1_-triggered evolved CDN “P”.

The enzyme-free CHA reaction for the emergence of CDNs was further applied to synthesize a complex [3 × 3] CDN “P”, as outlined in Figure 2. In this case, a set of six hairpin motifs H_A_–H_C1_ was employed as the parent reaction module. The primer-P_1_-activated CHA reaction resulted in the amplified generation of nine duplex constituents AA_1_, AB_1_, AC_1_, BA_1_, BB_1_, BC_1_, CA_1_, CB_1_, and CC_1_, leading to the high-throughput self-assembly of the [3 × 3] CDN “P”, Figure 2A. The time-dependent fluorescence changes generated by the nine DNAzyme reporter units, associated with the nine constituents of the CDN “P”, are shown in Figure 2B. Using the respective calibration curves that relate the cleavage rates of the substrates to the concentrations of the intact constituents, Figures S1 and S2, the concentrations of the constituents in the emerged [3 × 3] CDN “P” were assessed, and these are summarized in Table S2. A gel electrophoretic experiment was further performed to support the P_1_-guided evolution of the [3 × 3] CDN “P” and to evaluate the contents of the constituents in CDN “P”; see Figure S4 and the accompanying detailed discussion.

**Figure 3:**
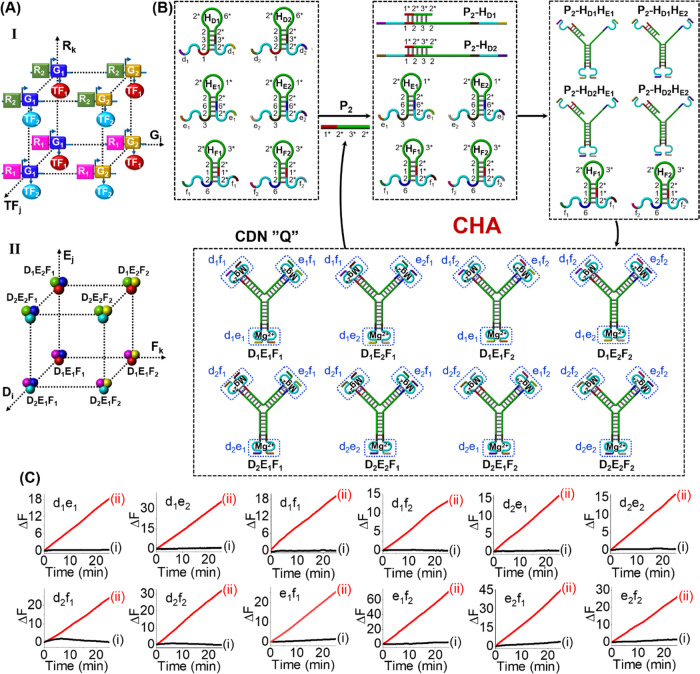
(A) Panel I: Gene regulatory network comprising intercommunicating constituents consisting of three components, e.g., the gene (G_i_), the transcription factor (TF_j_), and the regulating unit (R_k_). Panel II: An artificial equilibrated 3D CDN composed of eight equilibrated three-component constituents, D_i_E_j_F_k_. (B) Primer-P_2_-guided evolution of a 3D CDN “Q” using a set of six functional hairpin structures and the enzyme-free CHA reaction. (C) Time-dependent fluorescence changes generated by DNAzyme reporter units: (i) in the absence of primer P_2_, and (ii) in the presence of primer-P_2_-triggered evolved CDN “Q”.

All DNA-based CDN systems reported to date were composed of constituents consisting of two components. In nature, CDNs composed of three or more components play pivotal roles in the development of living systems.^56,57^ For instance, gene regulatory networks contain intercommunicating constituents, comprising three components, including the gene, G_i_, the transcription factor, TF_j_, and the regulating unit, R_k_, in the promoter domain (Figure 3A, panel I). The intercommunication between these three components leads to the diversity of upregulated or downregulated genes. Inspired by the natural network, the evolution of 3D CDNs composed of three components (D_i_, E_j_, F_k_) emulating native networks is designed by the catalytic assembly of a three-arm DNA junction using branched CHA. This is exemplified in Figure 3B with six properly designed hairpin units, H_D1_–H_F2_. The introduction of P_2_ into the system activates a cascade of assembly steps with H_Di_, H_Ej_, and H_Fk_. P_2_ opens the hairpins H_D1_ and H_D2_ to generate duplexes H_D1_–P_2_ and H_D2_–P_2_ acting as triggers to unfold hairpins H_E1_ and H_E2_, resulting in the formation of four transient intermediate triplex, H_D1_–H_E1_–P_2_, H_D1_–H_E2_–P_2_, H_D2_–H_E1_–P_2_, and H_D2_–H_E2_–P_2_. The newly generated triplex leads to the opening and hybridization of H_F1_ and H_F2_, followed by a disassembly step in which F_j_ displaces P_2_ from the complex, releasing P_2_ to catalyze the self-assembly of additional branched junctions. Thus, the primer P_2_ activates the CHA reaction, leading to the amplified generation of eight three-arm nucleic acid-based constituents, D_1_E_1_F_1_, D_1_E_1_F_2_, D_1_E_2_F_1_, D_1_E_2_F_2_, D_2_E_1_F_1_, D_2_E_1_F_2_, D_2_E_2_F_1_, and D_2_E_2_F_2_, comprising the dynamically equilibrated three-dimensional CDN “Q”, Figure 3A. Each of the constituents is functionalized with three DNAzyme units, acting as reporter units. Each of the reporter units cleaves the respective fluorophore/quencher-modified substrates, and the resulting fluorescence signal provides a readout signal. That is, the eight constituents in the network are anticipated to yield twelve different fluorescence signals. Figure 3C shows the time-dependent fluorescence changes generated by the reporter units associated with the 12 constituents of the CDN “Q” formed upon subjecting the set of six hairpins shown in Figure 3A to the primer P_2_. Under these conditions, all 12 DNAzyme reporter units were activated (Figure 3C), indicating the formation of an equilibrated 3D CDN “Q” system. Gel electrophoresis experiments confirmed that the hairpins could not assemble into a three-arm DNA junction in the absence of the primer P_2_ and that the hairpins were almost entirely converted into three-arm DNA junction structures by the addition of P_2_ (Figure S3). The evolution of the CDN “Q” by P_2_-triggered branched CHA and the quantitative evaluation of its constituents were further supported by a quantitative electrophoretic experiment, Figure S6. For a detailed description of the quantitative assessment of the contents of constituents in CDN “Q”, see Figure S6 and Tables S3.

**Figure 4:**
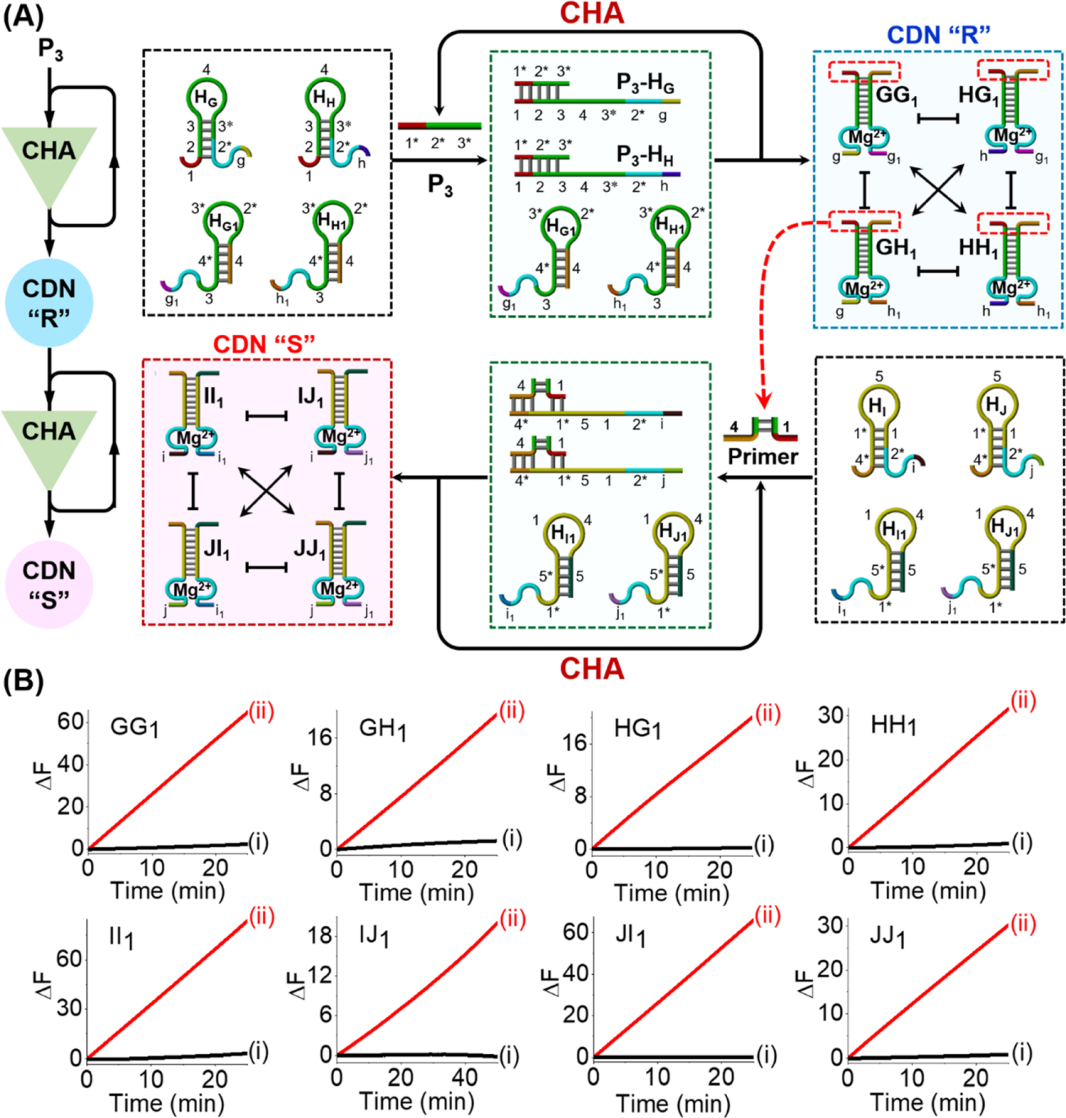
(A) Primer-P_3_-guided cascaded evolution of CDN “R” and CDN “S” using a set of eight functional hairpin structures and a two-layer CHA cascade. (B) Time-dependent fluorescence changes generated by DNAzyme reporter units: (i) in the absence of primer P_3_, (ii) in the presence of primer-P_3_-triggered cascaded evolution of CDNs “R” and “S”.

The emergence of CDNs of diverse compositions and complexities demonstrated [2 × 2] CDN, [3 × 3] CDN, and 3D CDN that were synthesized by the enzyme-free CHA process and appropriately engineered hairpin motifs. Inspired by nature, where environmentally triggered complex dynamic networks in evolution of life^38^ and cellular transformations involve signal-triggered feedback processes and cascaded reactions,^13,58^ we designed a two-layer cascaded emergence of CDNs employing a self-catalytic cascaded CHA circuit, as outlined in Figure 4A. In the parent system, eight hairpin motifs, H_G_, H_G1_, H_H_, H_H1_, H_I_, H_I1_, H_J_, and H_J1_, coexist as stable structures in the absence of primer P_3_. The primer P_3_ catalyzes the assembly of four hairpins, H_G_, H_G1_, H_H_, and H_H1_, to form four duplex constituents, GG_1_, GH_1_, HG_1_, and HH_1_, comprising CDN “R”. Concomitantly, P_3_ was regenerated to successively activate the first-layer CHA circuit, leading to the continuous assembly of CDN “R”. The formation of the four constituents through the first-layer CHA brings the separate single-stranded tethers 1 and 4 into close proximity, acting as a trigger to stimulate the second-layer CHA reaction. The colocalized tethers 1–4 open hairpins H_I_ and H_J_ and lead to the autonomous opening of H_I1_ and H_J1_, resulting in the generation of another four duplex constituents II_1_, IJ_1_, JI_1_, and JJ_1_ comprising CDN “S”. Meanwhile, tethers 1–4 could be separated, allowing the successive activation of second-layer CHA and the amplified generation of CDN “S”. Each of the constituents in CDNs “S” and “R” includes DNAzymes acting as reporter units to follow the quantitative evaluation of the contents of the constituents. Figure 4B shows the time-dependent fluorescence changes generated by the reporter units associated with the constituents of the CDNs “R” and “S” formed upon subjecting the set of hairpins shown in Figure 4A to the primer P_3_. All eight DNAzyme reporter units were activated (Figure 4B), indicating the cascaded emergence of two equilibrated CDNs “R” and “S”. A control experiment (Figure S7) revealed that subjecting the primer P_3_ to the separated second-layer CHA module did not activate DNAzyme units associated with CDN “S”, indicating that the catalytic assembly behavior of the system originates, indeed, from the cascaded intercommunication of two-layer CHA modules. Using the respective calibration curves that relate the rates of cleavage of the substrates to the different concentrations of the intact constituents (Figures S8 and S9), the concentrations of the constituents in the resulting evolved CDNs “R” and “S” were evaluated, and these are summarized in Table S4. Complementary quantitative electrophoretic separation of the constituents associated with the evolved CDNs “R” and “S” was performed to confirm the expected cascaded catalytic assembly behavior (Figure S10), and the corresponding results are summarized in Table S4. Very good agreement between the concentrations of the constituents determined by the DNAzyme reporter units and by the quantitative electrophoretic experiments is demonstrated. In addition, the concept of the nonenzymatic two-layer CHA cascaded amplification circuit and multiple fluorescence readout signals generated by DNAzyme reporter units associated with four constituents in evolved CDN “S” was applied to develop a universal sensing platform using the oncogene biomarker miRNA-21,^59^ as the model target (Figure S11 and accompanying discussion). The use of multiple synergistic fluorescence readout signals produced by four intercommunicating constituents of evolved CDN “S” provides a reliable method to avoid false-positive signals, and the two-layer CHA cascade amplification circuit reveals a low detection limit of 10 pM, accompanied by a sensitivity improvement of 2 orders of magnitude over single-layer CHA.

**Figure 5:**
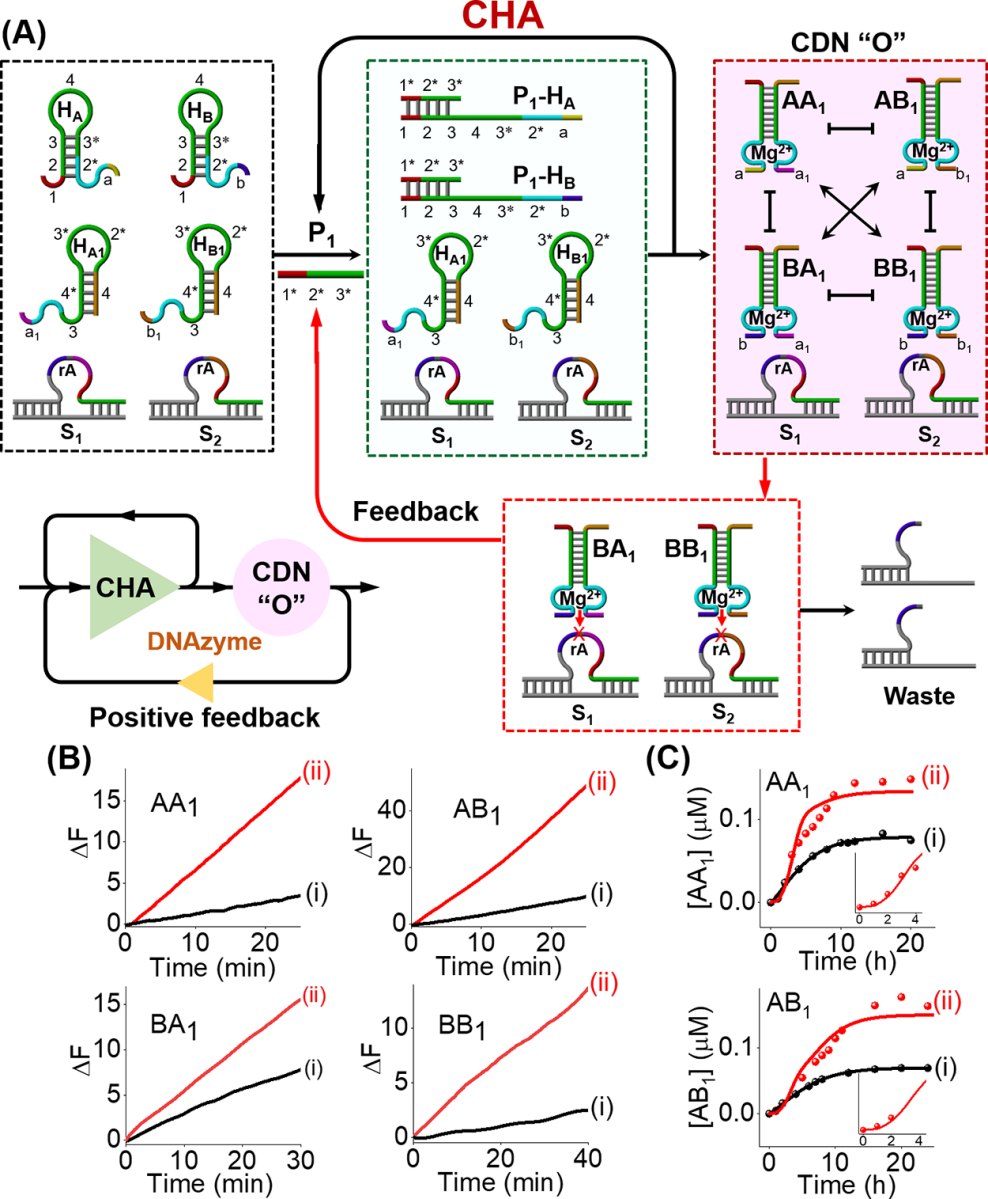
(A) Primer-P_1_-guided cross-catalytic feedback circuit for the emergence of CDN “O”. (B) Time-dependent fluorescence changes generated by DNAzyme reporter units after a time interval of 12 h: (i) in the presence of the P_1_-triggered nonfeedback circuit, and (ii) in the presence of the P_1_-triggered cross-catalytic feedback circuit. (C) Time-dependent concentration changes of the constituents AA_1_ and AB_1_ evolved in CDN “O”, dotted curves: (i) upon the P_1_-triggered nonfeedback driven circuit, and (ii) upon the P_1_-triggered cross-catalytic feedback-driven circuit. Insets: Nonlinear, concentration changes of the constituents at short time intervals. Solid curves overlayed on the experimental dotted curves correspond to the computationally simulated results using the kinetic model displayed in Figure S13.

In the next step, a feedback-driven emergence of CDN was designed using a cross-catalytic CHA–DNAzyme circuit comprising two catalytic assembly cycles, i.e., CHA and DNAzyme-powered positive-feedback loop, as displayed in Figure 5A. The CHA–DNAzyme circuit consists of four hairpins motifs, H_A_, H_A1_, H_B_, and H_B1_, and two ribonucleobase-containing caged substrates S_1_ and S_2_ that can be cleaved by respective DNAzymes to release the protected primer sequence P_1_. Subjecting the system to the primer P_1_ initiates the CHA reaction, leading to the continuous assembly of four duplex constituents AA_1_, AB_1_, BA_1_, and BB_1_ comprising CDN “O”. Each of these constituents includes a different DNAzyme unit. Subsequently, the DNAzymes associated with constituents BA_1_ and BB_1_ cleave the caged substrates S_1_ and S_2_, respectively, to continuously generate the protected P_1_ sequence that, in turn, catalyzes the CHA reaction. Thus, the DNAzyme-mediated positive-feedback loop provides an amplification path for the CHA for self-assembly of the constituents AA_1_, AB_1_, BA_1_, and BB_1_, thus realizing the isothermal feedback-driven emergence of CDN “O”. A nonfeedback circuit using non-ribonucleobase-containing substrates was also established to demonstrate the DNAzyme-mediated cross-catalytic feedback mechanism. Figure 5B shows the time-dependent fluorescence changes by CDN “O” formed by the cross-catalytic feedback circuit (curve (ii)) and by the nonfeedback circuit (curve (i)) after a time interval of 12 h. An evident fluorescence increase is shown in the primer-P_1_-triggered cross-catalytic feedback circuit, whereas a relatively low fluorescence increase is revealed in the P_1_-triggered nonfeedback circuit, indicating that the cleavage of substrates by DNAzyme is essential to evolve the effective formation of CDN “O”. We find that the concentration changes of the constituents in the CDN are slow compared with the cleavage rates of the substrates by the DNAzyme reporters. This allows us to monitor the compositions of the CDN at different time intervals until it reaches the final equilibrium. The catalyzed cleavage rates of the substrates associated with AA_1_ and AB_1_ increase during the feedback-driven evolution of CDN “O”, Figure S12. Figure 5C shows the quantitative concentrations of the constituents AA_1_ and AB_1_ at different time intervals during equilibration of CDN “O”. Evidently, the contents of AA_1_ and AB_1_ increase nonlinearly within a time interval of ca 12 h until they tend to reach saturation. The dynamic nonlinear evolution of CDN “O” was kinetically modeled, Figure S13. The experimental temporal concentrations of the constituents AA_1_ and AB_1_ (dotted curves, Figure 5C) were computationally simulated using a kinetic model. The computationally fitted temporal concentrations of AA_1_ and AB_1_ are overlayed as solid curves on the experimental data. The best-fit rate-constants of the stepwise reactions involved in the feedback-driven circuit are summarized in Table S5. In addition, the cross-catalytic feedback amplification system and multiple fluorescence readout signals generated by the evolved CDN “O” were, then, used to develop a universal sensing platform for miRNA-21 detection (Figure S14 and accompanying discussion). The feedback amplification system reveals a low detection limit of 1 pM, with sensitivity improvements of 3 orders of magnitude over the nonfeedback system.

**Figure 6:**
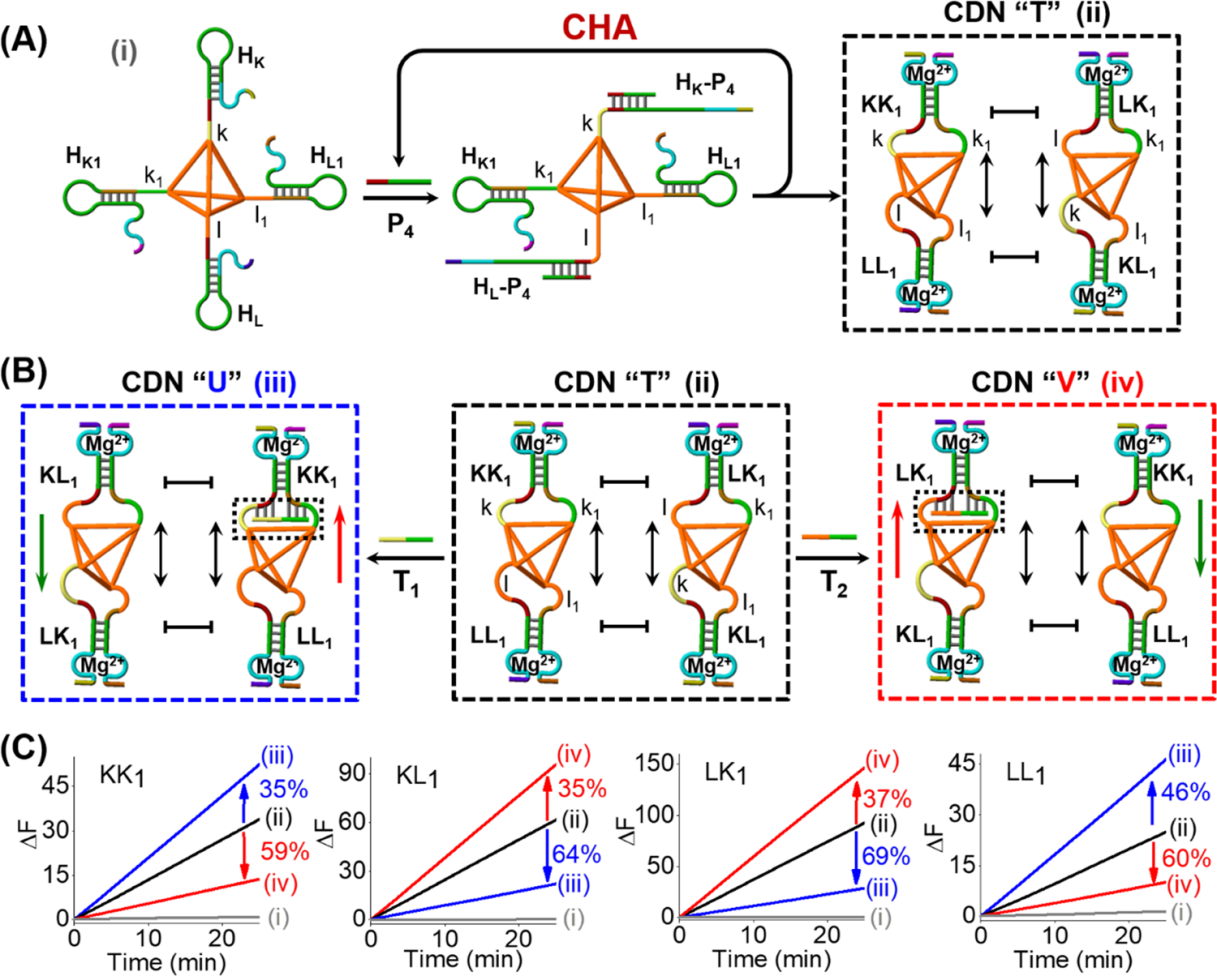
(A) Primer-P_4_-guided spatially localized CHA-stimulated, amplified emergence of CDN “T” using a DNA-hairpin-modified tetrahedron as the functional nanostructure and P_4_ as the trigger. (B) Schematic orthogonal reconfiguration of localized CDN “T” into “U” or “V” in the presence of the triggers T_1_ or T_2_. (C) Time-dependent fluorescence changes generated by DNAzyme reporter units: (i) in the absence of P_4_, (ii) upon P_4_-triggered evolved CDNs “T”, (iii) upon the T_1_-triggered reconfiguration of CDN “T” to CDN “U”, and (iv) upon the T_2_-triggered reconfiguration of CDN “T” to CDN “V”.

Theoretical models suggested that spatial organization of a dynamically interacting nucleic acid nanostructure within a “core” framework could enhance the reactivity of the intercommunicating nanostructures. Such supramolecular interactions could have important consequences in the evolution of life within an RNA world under prebiotic conditions.^37,38^ Besides, cells provide confined microenvironments in which intercommunication between networks and network-guided catalytic transformation leads to effective cell functions.^60−63^ The spatial confinement of the cell provides a means for effective signal transfer and communication between the networks and efficient network-guided activation of biocatalytic cascades overcoming diffusional barriers. Accordingly, the integration of synthetic networks into native cells, or alternatively, loading of cell-like containment with functional dynamic networks,^40,64−66^ is a major challenge in the field. DNA nanotechnology introduced nucleic acid nanostructures, such as origami rafts^8,67−69^ or DNA tetrahedra,^70,71^ that interact with cells or protocell assemblies. Indeed, DNA tetrahedra demonstrated effective permeation into cells as compared to duplex DNA.^72,73^ Thus, we argued that organization of a dynamically interacting hairpin nucleic acid nanostructure associated with “core” DNA-tetrahedra frameworks could emulate supramolecular interactions under prebiotic conditions. Particularly, the integration of these nanostructures into cellular containments could reveal the benefits of spatial confinement on the emergence of a dynamic network. Following this concept, we assembled a DNA tetrahedron with four engineered hairpin structures as a functional module that guides, in the presence of an appropriate strand, the CHA-induced evolution of a tetrahedra-based CDN that undergoes a switchable dynamic reconfiguration. We, then, adapted this concept to introduce the four-hairpin-functionalized DNA tetrahedron into cells, and spatial evolution of the dynamic network in the cells was activated by intracellular miRNA. Beyond the spatial intracellular evolution of the CDN, the process was applied to sense and image the native containment. This is schematically depicted in Figure 6A, where a DNA tetrahedron is functionalized at each of its corners with four appropriately engineered hairpin motifs H_K_, H_K1_, H_L_, and H_L1_, which include an extended single-strand tether for hybridization with the hairpin sequences. The DNA tetrahedron was purified to remove free hairpin structures, and gel electrophoresis confirmed the formation of the pure intact hairpin-functionalized tetrahedron (Figure S15 and accompanying discussion). Subjecting the hairpin-functionalized tetrahedron to P_4_ led to the opening of H_K_ and H_L_ and to the successive opening of H_K1_ and H_L1_, thus generating two constituent-functionalized tetrahedra: KK_1_-/LL_1_-functionalized tetrahedron and KL_1_-/LK_1_-functionalized tetrahedron. The four duplex structures are conjugated to single-stranded arms (“k”, “k_1_”, “l”, and “l_1_”) that provide hybridization domains for the respective triggers. These two tetrahedra can dynamically exchange their configuration, leading to the dynamically equilibrated CDN “T”. The orthogonal-triggered reconfiguration of the CDN “T” localized on tetrahedra proceeds in the presence of the triggers T_1_ or T_2_, Figure 6B. The subjecting of equilibrated CDN “T” to trigger T_1_ stabilizes KK_1_, leading to the upregulation of constituents KK_1_- and LL_1_-functionalized tetrahedra and to the concomitant downregulation of constituents KL_1_- and LK_1_-functionalized tetrahedra, resulting in the reconfiguration of CDN “T” to CDN “U”. The T_1_-triggered reconfiguration of CDN “T” to “U” was followed by the responses of the DNAzyme units associated with the constituents, Figure 6C. The time-dependent fluorescence changes of the DNAzyme reporter units associated with the constituents of the parent CDN “T” and CDN “U” generated, in the presence of T_1_, are displayed in curves (ii) and (iii), respectively. The reconfiguration of CDN “T” into CDN “U” is accompanied by the upregulation of KK_1_ and LL_1_ by 35 and 46% and the downregulation of KL_1_ and LK_1_ by 64 and 69%, respectively. Similarly, subjecting CDN “T” to trigger T_2_ leads to the reconfiguration of CDN “T” into CDN “V”, where the constituent LK_1_ is stabilized by T_2_. As a result, the concentrations of constituents KL_1_- and LK_1_-functionalized tetrahedra are upregulated, whereas the concentrations of constituents KK_1_- and LL_1_-functionalized tetrahedra are downregulated. The time-dependent fluorescence changes generated by DNAzyme reporter units associated with the constituents of the parent CDN “T” and CDN “V” are presented in Figure 6C, curves (ii) and (iv), respectively. The dynamic transition of CDN “T” into CDN “V” is accompanied by the upregulation of KL_1_ and LK_1_ by 35 and 37% and the downregulation of KK_1_ and LL_1_ by 59 and 60%, respectively. Using the respective calibration curves (Figures S16 and S17), the concentrations of the respective constituents in CDN “T”, CDN “U”, and CDN “V” were quantitatively evaluated, and the concentrations of the respective constituents are summarized in Table S6. Further quantitative gel electrophoresis experiments to evaluate the contents of the constituents associated with CDNs “T”, “U”, and “V” were performed (Figure S18), and the results are summarized in Table S6. Very good agreement between the contents of the constituents determined by the DNAzyme reporter units and by the quantitative electrophoretic experiments was obtained.

**Figure 7:**
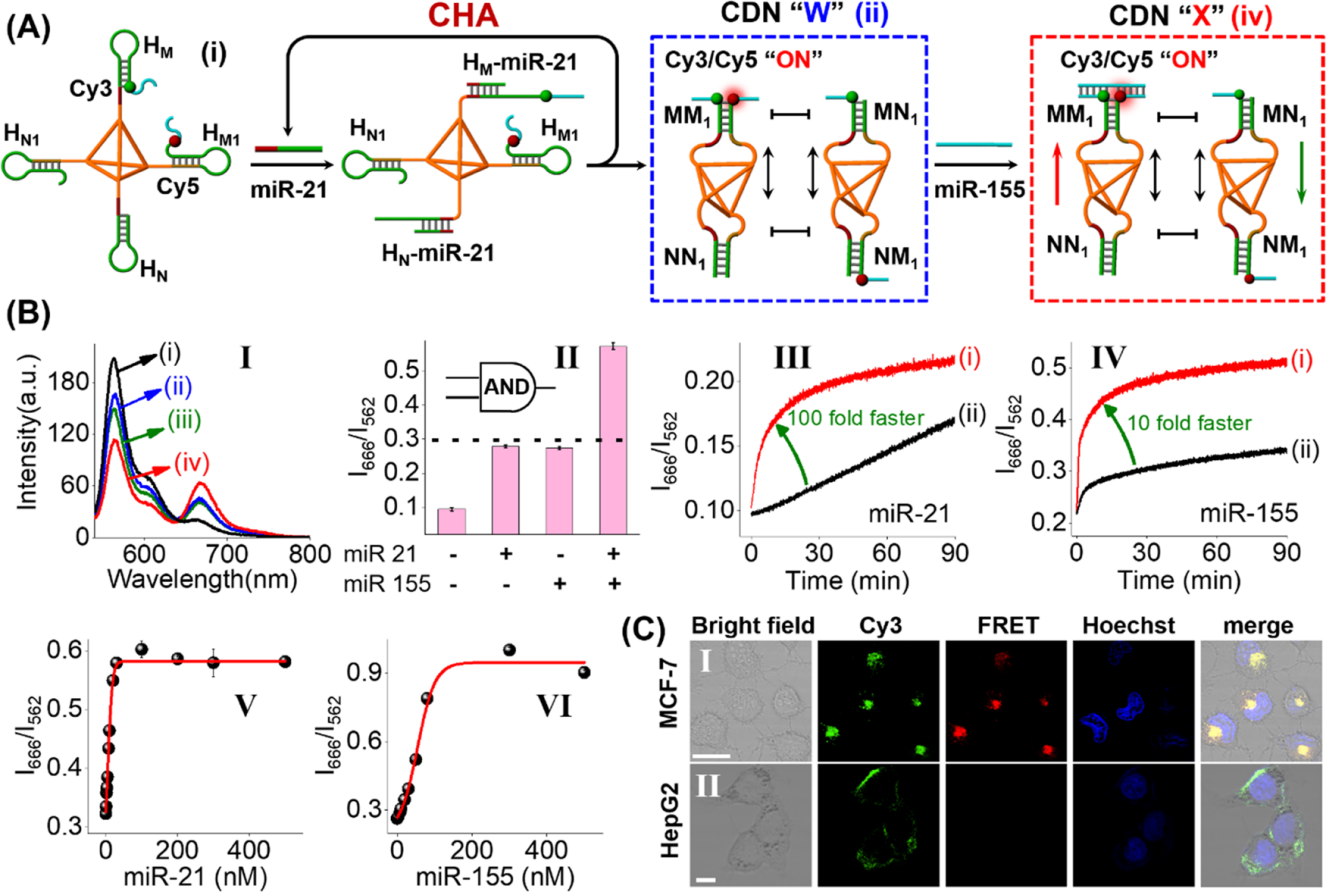
(A) Scheme of miRNA-triggered hierarchical adaptive control over the emergence and composition of CDN using localized CHA. (B) Logic-gated sensing of miRNA-21 and miRNA-155. Panel I: Fluorescence spectra of the localized CHA sensing system: (i) in the absence of miRNA-21 and miRNA-155, (ii) in the presence of miRNA-21, 50 nM, (iii) in the presence of miRNA-155, 50 nM, and (iv) in the presence of both miRNA-21 and miRNA-155, 50 nM each. Panel II: Display of the “AND” logic-gate sensing system in the form of a bar presentation. Panel III: Time-dependent FRET intensity changes of (i) miRNA-21-initiated localized CHA, and (ii) miRNA-21-initiated free CHA without a tetrahedron (diffusible components). Panel IV: Time-dependent FRET intensity change (i) upon the miRNA-155-triggered reconfiguration of localized CDN “W” into “X”, and (ii) upon the miRNA-155-triggered reconfiguration of CDN “W” into “X” using free CHA. Panel V: Calibration curve of miRNA-21, in the presence of miRNA-155, 50 nM. Panel VI: Calibration curve of miRNA-155, in the presence of 50 nM miRNA-21. Error bars derived from *N* = 3 experiments. (C) Bright-field microscopy images, confocal fluorescence microscopy images, and merged images corresponding to the analyses of (entry I) MCF-7 cells and (entry II) HepG2 cells treated with the hairpin-functionalized tetrahedron.

The effective nonenzymatic CHA-assisted, spatially localized, signal-amplified assembly of the CDN and the accompanying enhanced cell permeation properties of the DNA-tetrahedra nanostructures suggest that appropriately engineered hairpin tetrahedra could provide functional vehicles for the emergence of an intracellular network. Furthermore, the strand-induced reconfiguration of the CDN enables the application of an intracellular or nucleic acid biomarker as a trigger to reconfigure the CDN and, thus, allow the use of the dynamic process as a sensing or imaging platform for the cellular biomarker. This is exemplified in Figure 7A with the use of localized CHA on the tetrahedron and the miRNA-triggered hierarchical adaptive control over emergence and compositions of the CDN as an intelligent nanoplatform to construct a logic circuit for intracellular miRNA sensing and imaging.

Figure 7A depicts schematically the method to apply the hairpin-functionalized tetrahedron for the logic-gated analysis of two different miRNAs, e.g., miRNA-21 and miRNA-155. We selected miRNA-21 and miRNA-155 as targets to emphasize the selectivity of the sensing platform and its logical sensing capacity. miRNA-21 is an oncogene biomarker that is overexpressed in most tumors,^59^ and miRNA-155 is overexpressed in breast cancer cells.^74^ As shown in Figure 7A, the tetrahedron is functionalized at each of its corners with the four hairpin motifs H_M_, H_M1_, H_N_, and H_N1_, while two out of the four hairpin motifs, H_M_ and H_M1_, were modified with fluorophores Cy3 and Cy5, respectively. Subjecting the tetrahedron to miRNA-21 initiates the spatially localized CHA reaction to generate two tetrahedra, constituents MM_1_- and NN_1_-functionalized tetrahedra and constituents MN_1_- and NM_1_-modified tetrahedra, leading to self-assembly into the CDN “W”. The partial formation of the constituent MM_1_ is anticipated to bring spatial proximity between the two fluorophores, thus yielding a slightly increased FRET signal. The subsequent treatment of CDN “W” with miRNA-155 transforms CDN “W” into CDN “X”, where miRNA-155 binds to the arms of the duplex constituent MM_1_, Figure 7A. Stabilization of MM_1_ leads to the upregulation of this constituent, the downregulation of MN_1_ and NM_1_, and the concomitant upregulation of NN_1_. The upregulation of constituent MM_1_ is, then, anticipated to significantly intensify the FRET process between Cy3 and Cy5. These two miRNAs together activate the Cy3/Cy5 FRET signal, which is consistent with a two-input AND logic gate.

The miRNA-responsive hierarchical adaptive control over CDN by spatially localized-confined CHA was in vitro-investigated via monitoring the Cy3/Cy5 FRET signal under different conditions. Figure 7B, panel I, curve (i), shows that no FRET signal is observed on the hairpin-functionalized tetrahedron. The addition of miRNA-21 activated the localized CHA reaction, resulting in the amplified formation of CDN “W”. This process is supported by the slightly increased FRET signal between Cy3 and Cy5, Figure 7B, panel I, curve (ii). The equilibrated CDN “W” was, then, subjected to miRNA-155, which binds to the arms of the duplex constituent MM_1_, resulting in the reconfiguration of CDN “W” to CDN “X”. The stabilization and upregulation of MM_1_ lead to an obvious increase in the FRET signal (panel I, curve (iv)), demonstrating that the sensing of two miRNAs is, indeed, feasible. The control experiment probing the effect of miRNA-155 on the FRET signal revealed that slight FRET was observed. These results confirm the AND logic operation of the proposed sensing platform and its potential capacity for the intracellular detection of miRNA-21 and miRNA-155 (Figure 7B, panel II). (Note that the error bars for the FRET intensities *I*_666_ (Cy5)/*I*_562_ (Cy3) were derived from *N* = 3 experiments and the associated error bars are small due to the reliable ratio of output signals.)

To demonstrate the benefits of spatially localized CHA for emergence of CDN, we experimentally compared signal propagation between the CHA reaction localized on the tetrahedron and free CHA without the tetrahedron (diffusible components). Figure 7B, panel III, curve (i), depicts the time-dependent FRET intensity ratio of the two fluorophores, *I*_666_/*I*_562_, upon miRNA-21-triggered localized CHA-induced evolution of CDN “W”. A FRET signal rapidly emerges for the spatially localized CHA-induced formation of the tetrahedra CDN (*t*_1/2_ < 8 mins), while a relatively slow and weak FRET enhancement is observed for the free CHA system (Figure 7B, panel III, curve (ii)). Evidently, spatially confined CHA for emergence of CDN decreases the reaction time from hours to minutes, compared to the CHA reaction with diffusible components. Meanwhile, the FRET change of localized CHA was much higher than that of free CHA, revealing the higher reaction efficiency of localized CHA. A similar behavior was also observed for miRNA-155-triggered reconfiguration of localized CDN “W” into “X”, Figure 7B, panel IV. Reconfiguration of localized CDN “W” into “X” shows a faster kinetic rate and a higher hybridization efficiency compared to the counterpart with the CHA diffusional components. The remarkably enhanced reaction kinetics and the higher reaction efficiency by localized CHA are ascribed to the increased collision probability and enhanced local concentrations, indicating that the localized CHA stimulates enhanced signal amplification capacities and a higher detection sensitivity.^8,75^

Figure 7B, panel V, depicts the calibration curve corresponding to the fluorescence intensity ratios, *I*_666_/*I*_562_, in the presence of variable concentrations of miRNA-21 with 50 nM miRNA-155. As the concentration of miRNA-21 increases, the ratio values of fluorescence intensities at *I*_666_ (characteristic of Cy5)/*I*_562_ (characteristic of Cy3) are intensified. The detection limit for sensing miRNA-21 corresponds to 2 nM. Figure 7B, panel VI, depicts the calibration curve of miRNA-155 in the presence of 50 nM miRNA-21. The detection limit for sensing miRNA-155 corresponds to 1 nM. Figure S19 shows the selectivity features of the sensing module. At a concentration of 100 nM miRNAs, the signal transduced is approximately fourfold enhanced in the presence of both target miRNA-155 and miRNA-21 as compared to the set of foreign miRNAs. The successful selective in vitro analysis of miRNAs was then applied to image the intracellular miRNAs associated with the different cells. In this experiment, we selected MCF-7, breast cancer cells, and HepG2, liver cancer cells. While the MCF-7 cells include two typical miRNAs, e.g., miRNA-21 and miRNA-155, the HepG2 cells include predominantly miRNA-21. Figure 7C, entry I, shows the bright-field image and the fluorescence features of the MCF-7 cells treated with the tetrahedra. The fluorescence of Cy3 (green) and the FRET emission of Cy5 (red) are observed. The resulting yellow color in the merged image confirms the operation of the FRET process between Cy3 and Cy5 and the existence of miRNA-21 and miRNA-155 in the cells. Figure 7B, entry II, depicts the bright-field image and the fluorescence features of HepG2 cells. The red fluorescence of the FRET signal could not be detected, implying that no FRET process between Cy3 and Cy5 proceeds in the cells. The merged image confirms that only the green fluorescence is visible. The results indicate effective FRET/donor efficiencies for Cy5 fluorescence for the MCF-7 cells, while for HepG2, inefficient FRET/donor fluorescence of Cy5 is visible. The results are consistent with the overexpression of miRNA-21 and miRNA-155 in MCF-7 cells. It should be noted that the confocal fluorescence images corresponding to fluorescent tetrahedra internalized in MCF-7 and HepG2 cancer cells differ in their shapes and intensities. Indeed, it is established that the internalization of DNA tetrahedra is cell-line-dependent and their spatial distribution in the cytoplasm is different.^76^ The differences in the confocal fluorescence images are due to these parameters.

## Conclusions

The present study has introduced a nonenzymatic isothermal catalytic DNA circuit consisting of synthetic DNA hairpins as a versatile system for the emergent synthesis of CDNs. The processes enabled the amplified high-throughput emergence of CDNs, revealing variable complexities and biological features including the formation of [2 × 2] CDN, [3 × 3] CDN, and three-arm junction-based 3D CDN, as well as the cascaded and feedback-driven emergence of CDNs using the two-layered CHA cascade circuit and the cross-catalytic CHA circuit. In addition, a spatially localized architecture for fast emergence and reconfiguration of CDN was also constructed, which performed significantly faster than the system with diffusible components. Benefiting from the high cellular uptake efficiency of the DNA tetrahedron, a spatially localized architecture for hierarchical adaptive control over the emergence and reconfiguration of CDN was, then, used as a reliable platform for logic-gated miRNA imaging in living cells. Besides the contribution of the study to the fields of systems chemistry, sensor, and imaging, the concept can be extended to assemble networks, revealing enhanced complexities and functionalities, and particularly to develop important practical applications of systems. (i) The CHA circuits used in the present study provide “simple” examples to prove the concept of the enzyme-free evolution of CDN. Nonetheless, by applying the circular CHA cascade, three- or more-layered linear CHA cascade, four-arm branched CHA, and dendrimer-like CHA, CDNs of enhanced complexities and functionalities may be envisaged. Particularly, branched CHA-generated constituents are anticipated to evolve dynamic hydrogel matrices, revealing switchable stiffness properties for controlled drug release and self-healing applications.^59^ (ii) The present study used the primer-triggered CHA as the driving machinery to evolve the CDNs. Other enzyme-free catalytic DNA circuits such as the hybridization chain reaction and the entropy-driven catalytic circuit may be envisaged as alternative pathways to evolve CDNs. Besides the activation of the DNA machineries by nucleic acid triggers, engineering of hairpin constituents comprising of aptamer sequences leading to the operation of the polymerization processes by cellular metabolites using aptamer/ligand–metabolite complexes may be envisaged. (iii) The tetrahedron as a spatially localized nanoreactor could be replaced by DNA origami scaffolds where more DNA hairpins can be spatially positioned to evolve CDN of enhanced complexity and functionality including [3 × 3] CDN, 3D CDN, and cascaded evolution of CDN. (iv) Different applications of the spatially localized, CHA-stimulated formation of DNA-tetrahedra CDN in cells using a hairpin-functionalized constituent and cellular promoter triggers could find important therapeutic uses. The formation of an intracellular reconfigurable dynamic network could provide functional frameworks for programmable gene silence. (v) The different CHA-induced reaction frameworks, described in the study, were operated under optimized compositions comprising the different hairpins and triggering promoters. The formulation of kinetic models and computational simulations of the experimental kinetics characterizing the different systems could provide frameworks to further understand the systems. While such studies are beyond the scope of the present study, the potential of such kinetic analyses was exemplified in Figure S13 and the accompanying discussion. (vi) Furthermore, realizing that a “pool” of nucleic acids is considered the origin of primordial life (“RNA world”), the results presented in the paper may introduce mechanistic pathways for the development of functional and catalytic networks under prebiotic conditions.

## Supporting Information Available

Materials, characterizations, methods, DNA sequence, calibration curves, gel electrophoretic images and accompanying discussion, computational kinetic models, kinetic profile of feedback-driven CDN “O”, and Tables S1–S6 (PDF)
