# Supplementary material for: Cascaded, Feedback-Driven, and Spatially Localized Emergence of Constitutional Dynamic Networks Driven by Enzyme-Free Catalytic DNA Circuits
Source: J Am Chem Soc. 2023 May 31;145(23):12617–29. doi: 10.1021/jacs.3c02083 (PMC10273234; doi:10.1021/jacs.3c02083)
Supplement: Supplementary file 1 — ja3c02083_si_001.pdf [file ja3c02083_si_001.pdf]

Supporting Information

Cascaded, Feedback-Driven and Spatially-Localized Emergence of Constitutional Dynamic Networks Driven by Enzyme-Free Catalytic DNA Circuits

Zhixin Zhou,*[§,†] Nina Lin,[§,†] Yu Ouyang,[‡] Songqin Liu,[†] Yuanjian Zhang,*[,†] and Itamar Willner*[,‡]

[†]School of Chemistry and Chemical Engineering, Southeast University, Nanjing 211189, China

[‡]Institute of Chemistry, The Hebrew University of Jerusalem, Jerusalem 91904, Israel

[§]These authors contributed equally

*E-mail: zhixin.zhou@seu.edu.cn

*E-mail: Yuanjian.zhang@seu.edu.cn

*E-mail: willnea@vms.huji.ac.il

Experiment Section

Materials. Tris-Acetate-EDTA (TAE) buffer solution, DNA Marker A (25-500 bp), and "GelRed nucleic acid gel stain" were purchased from Sangon Biotech Co., Ltd. (Shanghai, China). Agarose was purchased from Aladdin Reagent Co., Ltd. (China). Polyacrylamide gel electrophoresis (PAGE) Gel Fast Preparation Kits (15% and 7.5%) were purchased from Shanghai Epizyme Biomedical Technology Co., Ltd (China). Ultrapure water (resistance > 18 MΩ·cm) was used in all of the experiments. All DNA and RNA oligonucleotides were synthesized by Sangon Biotech Co., Ltd.

The oligonucleic acid sequences used in the study include (from 5' end to 3' end):

H_{A}: *TGATAAGATGGGTGTAGGAAG*TGCCACAAGTACACTTCCTACACCCATGTTTAGCTT

H_{A1}: ACCATTCAGCGATTAGGAAGTGTACTTGTGGCACTTCCTACACCCATTGCCACAAGTACA

H_{B}: *TGATAAGATGGGTGTAGGAAG*TGCCACAAGTACACTTCCTACACCCATGTTTCAGTT

H_{B1}: TGTCCTCAGCGATTAGGAAGTGTACTTGTGGCACTTCCTACACCCATTGCCACAAGTACA

H_{C}: *TGATAAGATGGGTGTAGGAAG*TGCCACAAGTACACTTCCTACACCCATGTTCCTGA

H_{C1}: CTGTTCAGCGATTAGGAAGTGTACTTGTGGCACTTCCTACACCCATTGCCACAAGTACA

H_{D1}: GATATCAGCGAT*GGTTGATGGGTGAGTGAATGGGTG*CACCCATAAGACCACCCATTCACTCACCCATGTTTAGCT

H_{D2}: CTGCTCAGCGAT*GGTTGATGGGTGAGTGAATGGGTG*CACCCATAAGACCACCCATTCACTCACCCATGTTTCAGT

H_{E1}: CCATTCAGCGATAGTGAATGGGTGGTCTTATGGGTGCACCCATCAACCCACCCATAAGACCACCCATGTTTCAGT

H_{E2}: GTCCTCAGCGATAGTGAATGGGTGGTCTTATGGGTGCACCCATCAACCCACCCATAAGACCACCCATGTTCCTGA

H_{F1}: CTGTTCAGCGATGTCTTATGGGTGGGTTGATGGGTGCACCCATTCACTCACCCATCAACCCACCCATGTTACTCT

H_{F2}: GTCCTCAGCGATGTCTTATGGGTGGGTTGATGGGTGCACCCATTCACTCACCCATCAACCCACCCATGTTCGTCA

H_{G}: AGCA*TGATAGAATGGGTGTAGGAAG*CACCCATACAATGCTTCCTACACCCATGTTACTCT

H_{G1}: GATATCAGCGATTAGGAAGCATTGTATGGGTGCTTCCTACACCCATCACCCATACAATGG

H_{H}: AGCA*TGATAGAATGGGTGTAGGAAG*CACCCATACAATGCTTCCTACACCCATGTTCGTCA

H_{H1}: CTGCTCAGCGATTAGGAAGCATTGTATGGGTGCTTCCTACACCCATCA CCCATACAATGG

H_I: CCATTGTATGGGTGTCTATCATGCTAGACGTACATGATAGACACCCATGT TTCAGT

H_{I1}: CTGTTCAGCGATTCTATCATGTACGTCTAGCATGATAGACACCCATACAT GCTAGACGTACA

H_J: CCATTGTATGGGTGTCTATCATGCTAGACGTACATGATAGACACCCATGT TCCTGA

H_{J1}: GTCCTCAGCGATTCTATCATGTACGTCTAGCATGATAGACACCCATACA TGCTAGACGTACA

H_K: AGAATCTGATAGAGGTAGAG*TGATTGATAAGATGGGTGTAGGAAG*TGCCA CAAGTACACTTCCTACACCCATGTTCGTCA

H_{K1}: GATATCAGCGATTAGGAAGTGTACTTGTGGCACTTCCTACACCCATTG CCACAAGTACAAAATGTAGTGCTTACGTCAGGCAG

H_L: CTTACGTAGTTAGTATGCTTGCTGT*TGATAAGATGGGTGTAGGAAG*TGCC ACAAGTACACTTCCTACACCCATGTTACTCT

H_{L1}: CTGCTCAGCGATTAGGAAGTGTACTTGTGGCACTTCCTACACCCATTG CCACAAGTACAGGCAGACCAGAGTCTTGTACCAGT

H_M: AGAATCTGATAGAGGTTTTTCAACATCAGTCTGATAAGCTACCATGTG TAGATAGCTTA-Cy3-TCAGACTGATTAGCATTAA

H_{M1}: GCTTACGTCAGGCAGTTTTACCCCTATCAC-Cy5-TTAAGCTATCTACA CATGGTAGCTTATCAGACTCCATGTGTAGA

H_N: CTTACGTAGTTAGTATTTTTTCAACATCAGTCTGATAAGCTACCATGTG TAGATAGCTTATCAGACT

H_{N1}: GACTCTTGTACCAGTTTTTTTAAGCTATCTACACATGGTAGCTTATCA GACTCCATGTGTAGA

DNA sequences for assembly of substrates S_1 and S_2 shown in Figure 5:

S_1: CTTCCTACACCCATCTTATCAACTGAATrAGGAGGACCATCGACCATCAG TTTCACAGAC

S_2: CTTCCTACACCCATCTTATCAACTGAATrAGGAATGGCATCGACCATCAG TTTCACAGAC

L: GTCTGTGAAACTGATGGTCGATGTTAGTTGGCTGTAGGAAG

DNA sequences for assembly of DNA tetrahedron:

S_K/S_M: CCTCTATCAGATTCTTTACATTCCTAAGTCTGAAACATTACAGCTTG CTACACGAGAAGAGCCGCCATAGTA

S_L/S_N: TACTAACTACGTAAGTATTATCACCAGGCAGTTGACAGTGTAGCAAG CTGTAATAGATGCGAGGGTCCAATAC

S_{K1}: TCAACTGCCTGGTGATAAAACGACACTACGTGGGAATCTACTATGGCG GCTCTTCTTTCTGCCTGACGTAAGC

S_{L1}: TTCAGACTTAGGAATGTGCTTCCCACGTAGTGTCGTTTGTATTGGACCC TCGCATTTTACTGGTACAAGACTC

S_{M1}: CTGCCTGACGTAAGCTTTTCAACTGCCTGGTGATAAAACGACACTACG TGGGAATCTACTATGGCGGCTCTTC

S_{N1}: ACTGGTACAAGAGTCTTTTTTTCAGACTTAGGAATGTGCTTCCCACGTA

GTGTCGTTTGTATTGGACCCTCGCAT
H_{P1}: TCAGACTGATGTTGATGCTTCCTACACCCATCTTATCATCAACATCAGT CTGATAAGCTA
H_{P2}: TCAGACTGATGTTGATGCTTCCTACACCCATTCTATCATCAACATCAGT CTGATAAGCTA
P_1: CTTCCTACACCCATCTTATCA
P_2: CACCCATTCACTCACCCATCAACC
P_3: CTTCCTACACCCATTCTATCA
P_4: CTTCCTACACCCATCTTATCA
T_1: ACTACATTTTGTACTATCAATCACTCTA
T_2: ACTACATTTTGTACTATCAACAGCAAGCA
miRNA-221: 5'-AGCUACAUUGUCUGCUGGGUUUC-3'
miRNA-21: 5'-UAGCUUAUCAGACUGAUGUUGA-3'
miRNA-155: 5'-UUAAUGCUAAUCGUGAUAGGGGU-3'
miRNA-16: 5'-UAGCAGCACGUAAAUAUUGGCG-3'
DNA sequences for gel electrophoresis:
H_A-gel (2×2 CDN): TGATAAGATGGGTGTAGGAAGTGCCACAAGTACACTTCC TACACCCATGTTCGTCACACACACACACACACACACA
H_A-gel (3×3 CDN): TGATAAGATGGGTGTAGGAAGTGCCACAAGTACACTTCC TACACCCATGTTCGTCACACACACACACACACACACACACACACACA
H_{A1}-gel (2×2 CDN): ATTAGGAAGTGTACTTGTGGCACTTCCTACACCCATTGC CACAAGTACA
H_{A1}-gel (3×3 CDN): CGATTAGGAAGTGTACTTGTGGCACTTCCTACACCCATT GCCACAAGTACA
H_B-gel (2×2 CDN):TGATAAGATGGGTGTAGGAAGTGCCACAAGTACACTTCC TACACCCAT
H_B-gel-1 (3×3 CDN): TGATAAGATGGGTGTAGGAAGTGCCACAAGTACACTT CCTACACCCATGTTACTCTCTCTCTCTCT
H_B-gel-2 (3×3 CDN): AAAAAAAAAAAAAAAAAAAAAAAAAAAAAAAAAAAA AAAAAAAAAAAAAAAAAAAGAGAGAGAGAGAGTAAC
H_{B1}-gel (3×3 CDN): TCAGCGATTAGGAAGTGTACTTGTGGCACTTCCTACACC CATTGCCACAAGTACA
H_{D1}-gel: TCTCTCTCTATCAGCGATGGTTGATGGGTGAGTGAATGGGTGCACC CATAAGACCACCCATTCACTCACCCATGTTTAGCTCTCT
H_{D2}-gel: CTCTCTCTCTCTGCTCAGCGATGGTTGATGGGTGAGTGAATGGGTG CACCCATAAGACCACCCATTCACTCACCCATGTTTCAGTCTCTCTCTCT
H_{E2}-gel: CTCTCTCTCTGTCCTCAGCGATAGTGAATGGGTGGTCTTATGGGTG CACCCATCAACCCACCCATAAGACCACCCATGTTCCTGACTCTCTCTCT
H_{F1}-gel: CTCTCTCTCTCTGTTCAGCGATGTCTTATGGGTGGGTTGATGGGTG CACCCATTCACTCACCCATCAACCCACCCATGTTACTCTCTCTCTCTCT
H_{F2}-gel: GTCTTATGGGTGGGTTGATGGGTGCACCCATTCACTCACCCATCAA CCCACCCAT
H_G-gel: AGCATGATAGAATGGGTGTAGGAAGCACCCATACAATGCTTCCTAC ACCCATGTTACTCTCTCTCTCTCT

H_{H1}-gel: CTCTCTCTCTCTCTCTCTCTCTGCTCAGCGATTAGGAAGCATTGTAT GGGTGCTTCCTACACCCATCACCCATACAATGG
H_I-gel: CCATTGTATGGGTGTCTATCATGCTAGACGTACATGATAGACACCCA TGT
H_{I1}-gel: TCTATCATGTACGTCTAGCATGATAGACACCCATACATGCTAGACGT ACA
H_L-gel: TGCTTGCTGTGATAAGATGGGTGTAGGAAGTGCCACAAGTACACTT CCTACACCCATGT
H_{L1}-gel: AGCGATTAGGAAGTGTACTTGTGGCACTTCCTACACCCATTGCCAC AAGTACAGGCAGACCAGAGTCTTGTAC
Substrate sequences for DNAzyme subunits associated with consitituents:
Sub 1 (GG_1, d_1f_1, LK_1): ROX-AGAGTATrAGGATATC-BHQ2
Sub 2 (HH_1, d_2f_2, KL_1): ROX-TGACGATrAGGAGCAG-BHQ2
Sub 3 (GH_1, d_2f_1, LL_1): Cy5-AGAGTATrAGGAGCAG-BHQ2
Sub 4 (HG_1, d_1f_2, KK_1): FAM-TGACGATrAGGATATC-BHQ1
Sub 5 (II_1, BC_1, e_1f_1): FAM-ACTGAATrAGGAACAG-BHQ1
Sub 6 (JJ_1, CB_1, e_2f_2): ROX-TCAGGATrAGGAGGAC-BHQ2
Sub 7 (IJ_1, BB_1, d_2e_2): Cy5-ACTGAATrAGGAGGAC-BHQ2
Sub 8 (JI_1, CC_1, e_2f_1): FAM-TCAGGATrAGGAACAG-BHQ1
Sub 9 (D_1E_1, AA_1, d_1e_1): FAM-AGCTAATrAG GAATGG-BHQ1
sub10 (AC_1): Cy5-AGCTAATrAGGAACAG-BHQ2
sub11 (CA_1): ROX-TCAGGA TrAG GAATGG-BHQ2
sub12 (AB_1, d_1e_2): FAM-AGCTAATrAGGAGGAC-BHQ1
sub13 (BA_1): ROX-ACTGAATrAGGAATGG-BHQ2

The respective DNAzyme subunit sequences in each of hairpins are underlined. The sequences for recognition of primer (P_1, P_2, P_3 and P_4) are italic.

Measurements. Fluorescence spectra were recorded with a Cary Eclipse Fluorometer (Agilent Technologies). The excitation of FAM, ROX, and Cy5 was performed at 496 nm, 588 nm, and 648 nm, respectively, while the emission of FAM, ROX, and Cy5 was recorded at 516 nm, 608 nm, and 668 nm, respectively. The FRET fluorescence spectra between Cy3 and Cy5 were collected from 540 to 800 nm with excitation wavelength at 520 nm. UV/vis spectra were performed on a Cary 60 UV/Vis spectrometer (Agilent Technologies). The PAGE and agarose gels were run on VE-180 and HE-120 electrophoresis units, respectively (Tanon, China). The fluorescence of tetrahedra in cells was monitored with the Olympus FluoViewTM FV1000 confocal laser-scanning microscope, and all images were analyzed with image J.

Emergence of various DNA-based constitutional dynamic networks (CDNs) by catalytic hairpin assembly (CHA). The CHA assays were operated in 1 × PB buffer containing 10 mM NaH_2PO_4/Na_2HPO_4 (pH 7.0) and 20 mM $MgCl_2$, unless otherwise specificized.

For emergence of a [2×2] CDN "O" composed of AA_1, AB_1, BA_1 and BB_1, four hairpins, H_A, H_{A1}, H_B, and H_{B1}, 1 μM each, were mixed, and then the primer P_1, 1 μM or 10 nM, was subjected to the mixture, followed by incubating at 25 °C for 1 hour. The control experiment was performed under the same condition yet in the absence of P_1.

For emergence of a [3×3] CDN "P" composed of nine constituents, AA_1, AB_1, AC_1, BA_1, BB_1, BC_1, CA_1, CB_1, and CC_1, six hairpins, H_A, H_{A1}, H_B, H_{B1}, H_C, and H_{C1}, 1 μM each, were mixed, and then the primer P_1, 1 μM, was subjected to the mixture followed by incubating at 25 °C for 1 hour. The control experiment was performed under the same condition yet in the absence of P_1.

For emergence of a three-dimensional (3D) CDN "Q" composed of eight constituents, $D_1E_1F_1$, $D_1E_1F_2$, $D_1E_2F_1$, $D_1E_2F_2$, $D_2E_1F_1$, $D_2E_1F_2$, $D_2E_2F_1$, and $D_2E_2F_2$, six hairpins, H_{D1}, H_{D2}, H_{E1}, H_{E2}, H_{F1}, and H_{F2}, 1 μM each, were mixed, and then the primer P_2, 1 μM, was subjected to the mixture followed by incubating at 25 °C for 1 hour. The control experiment was performed under the same condition yet in the absence of P_2.

For cascaded emergence of CDN "R" and CDN "S", eight hairpins, H_G, H_{G1}, H_H, H_{H1}, H_I, H_{I1}, H_J, and H_{J1}, 1 μM each, were mixed, and then the primer P_3, 1 μM, was subjected to the mixture followed by incubating at 25 °C for 3 hours. The control experiment was performed under the same condition yet in the absence of P_3.

For feedback-driven emergence of CDN "O", four hairpins, H_A, H_{A1}, H_B, and H_{B1}, and caged substrates S_1 and S_2 were mixed, and the primer P_1, 10 nM, was subjected to the mixture followed by incubating at 30 °C. The non-cross-catalytic feedback circuit using non-ribonucleobase-containing substrates was performed under the same condition.

For emergence of CDN "T" attached on tetrahedra, the mixtures of S_K, S_{K1}, S_L, S_{L1}, H_K, and H_L, 1 μM each, were heated at 95 °C for 5 min and cooled to 4 °C within 1 min.

Subsequently, 1.5 μM H_{K1}, and H_{J1}, were incubated with the as-prepared H_K-/H_L-functionalized tetrahedron at 37 °C for 1 hour followed by purifying by ultrafiltration (30 kDa molecular weight cutoff) to remove the excessive H_{K1}, and H_{L1} remaining in solution. The primer P_4, 1 μM, was subjected to the mixture followed by incubating at 25 °C for 3 hours. The control experiment was performed under the same condition yet in the absence of P_4.

Methods

Evaluation of the concentrations of the constituents in the CDNs. Taking CDN "O" as an example, 100 μL of as-prepared equilibrated CDN mixture was treated with the corresponding fluorophore/quencher substrate (sub). As an example, to probe constituent AB_1 in CDN "O", 100 μL of the equilibrated CDN mixture was treated with the sub 12 (5 μL of 100 μM). Subsequently, the time-dependent fluorescence changes generated from the cleavage of sub 12 by the Mg^{2+}-dependent DNAzyme associated with the AB_1 were followed at 25 °C. Using the appropriate calibration curve corresponding to the rates of cleavage of the different substrates by different concentrations of the intact constituent (see detailed description in Figures S1 and S2), the contents of the constituent AB_1 in the different CDN "O" were evaluated. It should be noted that the concentrations of the constituents were not evaluated in a single step, but followed separately, according to this procedure for each of the constituents using the appropriate F_i/Q_i-substrate and the relevant calibration curve. The changes in the fluorescence intensities, as a result of the DNAzyme catalyzed cleavage of the respective substrates, ΔF, correspond to the measured fluorescence values, F_i, from which the background fluorescence of the respective fluorophore associated with the substrate, F_0, was subtracted.

Fluorescence Assay. For detection of miRNAs using localized CHA system, different concentrations of miRNA-21 and miRNA-155 were added to 100 μL of 1 × PB buffer solution containing 90 nM tetrahedron sensing module, followed by incubation for 5 hours at room temperature. The FRET fluorescence spectra between Cy3 and Cy5 were

collected from 540 to 800 nm with excitation wavelength at 520 nm.

Cell culture. Human breast cancer cells (MCF-7) were grown in 5% CO_2 DMEM medium supplemented with 10% FCS and 1% antibiotics (KeyGEN BioTECH, China). Human liver cancer cells (HepG2) were grown in 5% CO_2 DMEM medium supplemented with 10% FCS and antibiotics (KeyGEN BioTECH, China). Cells were planted one day prior to the experiment on μ-slide 4 well glass bottom for confocal microscopy.

Confocal microscopy measurements. For cell imaging experiments, one day prior to the experiment, cells were planted in μ-slide 4 well glass bottom. Cells were incubated with the tetrahedral DNA nanostructures after washing with PBS. The tetrahedra (100 nM) were incubated with cells for 7 hours and then washed with DMEM-Hepes twice and replenished with the fresh medium for the measurement. An external 561 nm excitation with an accompanying emission ranging from 570 to 620 nm was selected for the green channel of fluorophore (Cy3) donor. The external 561 nm FRET stimulation with an accompanying emission signal collection ranging from 650 to 700 nm was selected for the yellow channel of fluorophore (Cy5) acceptor. To achieve a reliable FRET readout, the background FRET signal, originating from solely Cy3/Cy5 fluorophore, was subtracted from each of the samples.

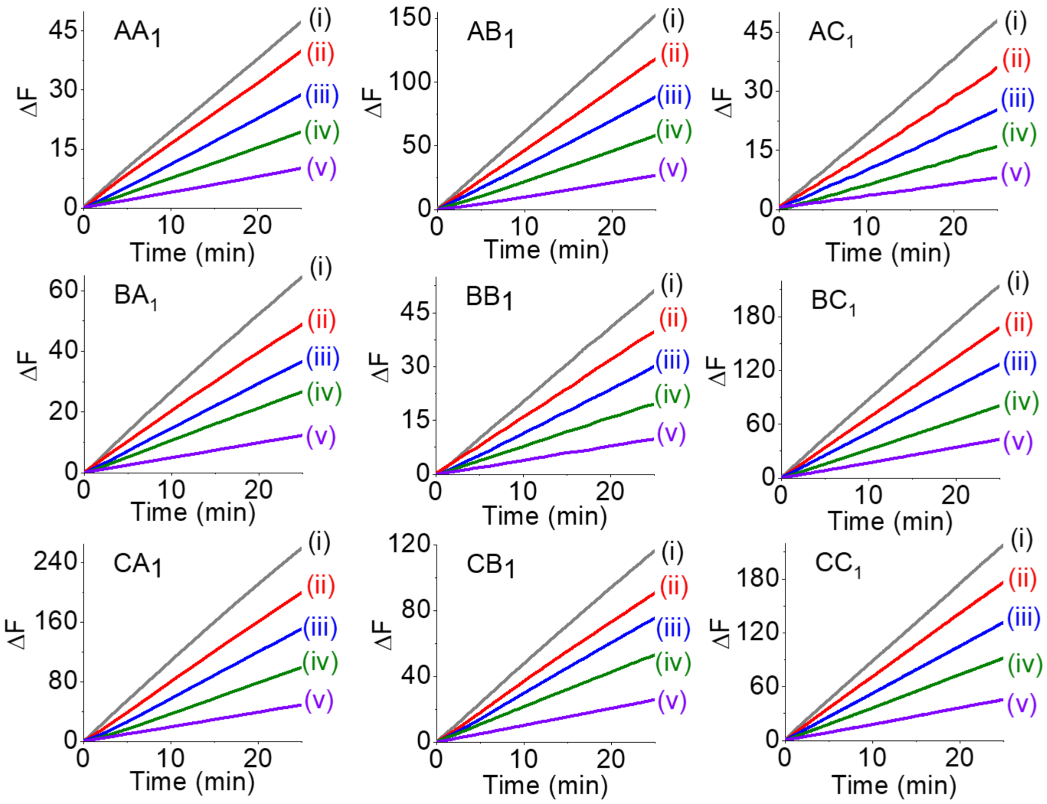

Figure S1. Time-dependent fluorescence changes generated upon the cleavage of the fluorophore/quencher-modified substrates by the respective Mg^{2+}-ion-DNAzyme reporter units associated with the individual intact constituents at variable concentrations: (i) 1 μM, (ii) 0.8 μM, (iii) 0.6 μM, (iv) 0.4 μM, and (v) 0.2 μM.

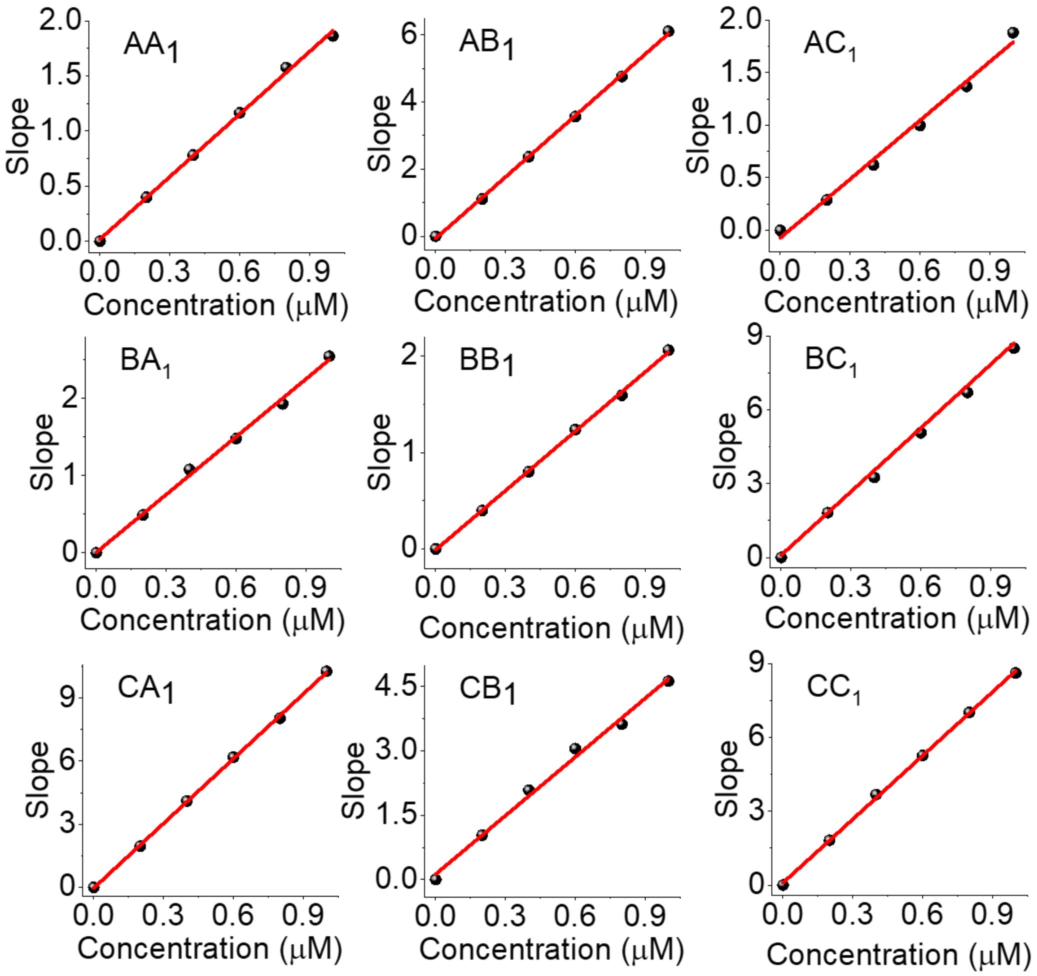

Figure S2. Corresponding calibration curves of the catalytic rates of the different constituents as a function of their concentrations, derived from the data shown in **Figure S1**.

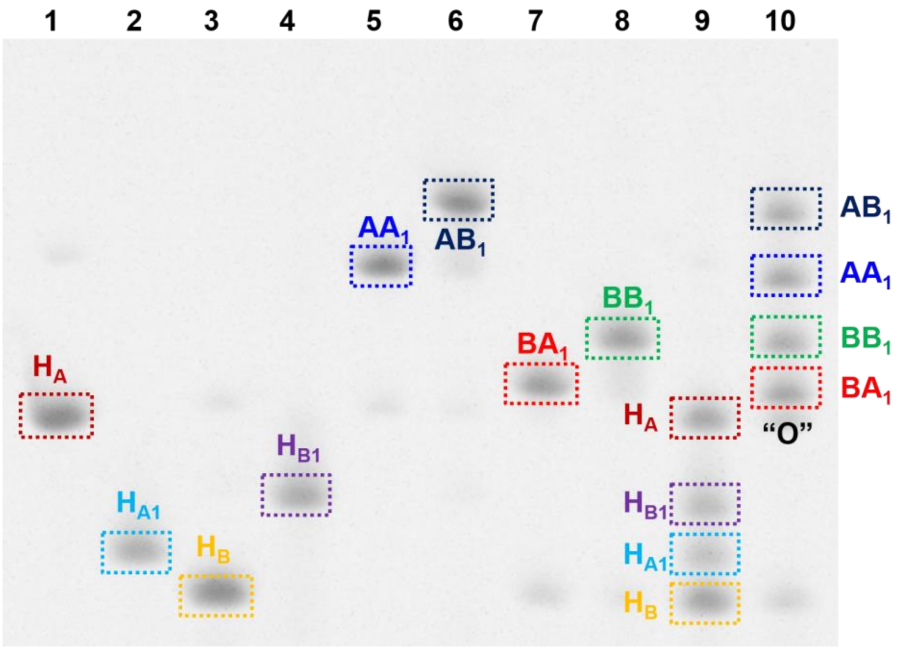

Figure S3. Gel electrophoresis (15% PAGE) demonstrating primer-P_1-guided evolution of CDN "O". For comparison, the bands of the individual hairpins, namely, H_A (lane 1), H_{A1} (lane 2), H_B (lane 3), and H_{B1} (lane 4), and the separated intact constituents, i.e., AA_1 (lane 5), AB_1 (lane 6), BA_1 (lane 7), and BB_1 (lane 8) are provided. Lane 9 corresponds to the mixture of four hairpins, H_A-H_{B1} in the absence of primer P_1. Lane 10 shows the separated constituents of CDN "O", generated upon the treatment of four hairpins with P_1. The results shown in lane 10 demonstrate the P_1-guided emergence of the CDN "O" comprising of the constituents AA_1, AB_1, BA_1, and BB_1.

From the intensities of the stained separated bands for evolved CDN "O" shown in lane 10 and using the stained bands of the individual intact constituents AA_1, AB_1, BA_1 and BB_1 at known concentrations (1 μM), we evaluated, using Image J software, the contents of the constituents in CDN "O", Table S1. As expected, we find that the contents of the constituents in the electrophoretically separated mixture of CDN "O" is similar to those evaluated by the DNAzyme reporter units, cf. Table 1 in the text.

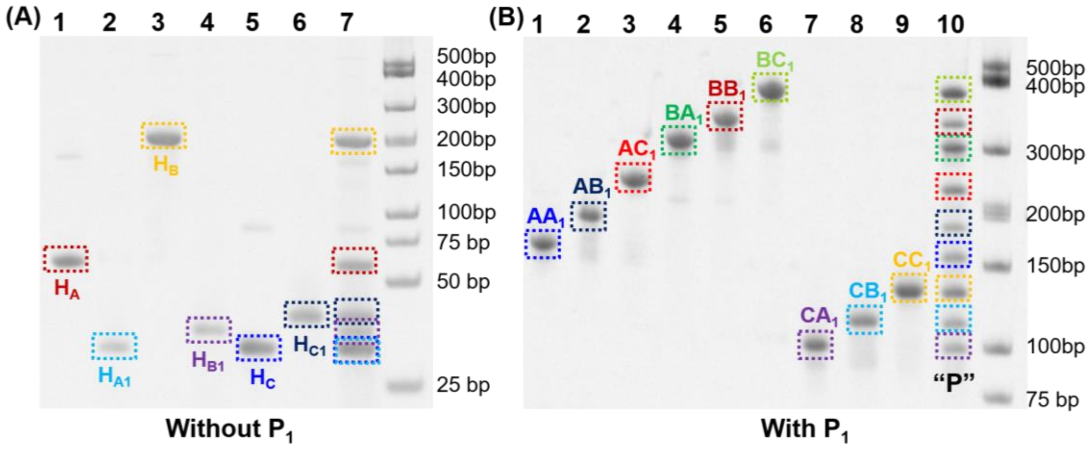

Figure S4. Gel electrophoresis (15% PAGE) demonstrating primer-P_1-guided evolution of CDN "P". (A) Gel electrophoretic image of the set of six hairpins, H_A-H_{C1} in the absence of P_1. Lane 1: H_A, lane 2: H_{A1}, lane 3: H_B, lane 4: H_{B1}, lane 5: H_C, lane 6: H_{C1}, lane 7: the mixture of six hairpins in the absence of P_1. (B) Gel electrophoretic image of the separated constituents generated, upon the primer-P_1-induced emerged formation of CDN "P" displayed in Figure 2. Lanes 1-9 correspond to the stained intact constituents associated with CDN "P": lane 1: AA_1, lane 2: AB_1, lane 3: AC_1, lane 4: BA_1, lane 5: BB_1, lane 6: BC_1, lane 7: CA_1, lane 8: CB_1, and lane 9: CC_1. Lane 10: the separated bands of CDN "P", generated upon the treatment of hairpins, H_A-H_{C1} with P_1. The results shown in lane 10 demonstrate the P_1-guided emergence of the CDN "P" comprising of the nine constituents AA_1, AB_1, AC_1, BA_1, BB_1, BC_1, CA_1, CB_1 and CC_1.

From the intensities of the stained separated bands for evolved CDN "P" shown in lane 10, Figure S4B, and using the stained bands of the individual intact constituents AA_1, AB_1, AC_1, BA_1, BB_1, BC_1, CA_1, CB_1, and CC_1 at known concentrations (1 μM), we evaluated, using Image J software, the contents of the constituents in CDN "P", Table S2. As expected, we find that the contents of the constituents in the electrophoretically separated mixture of CDN "P" is similar to those evaluated by the DNAzyme reporter units.

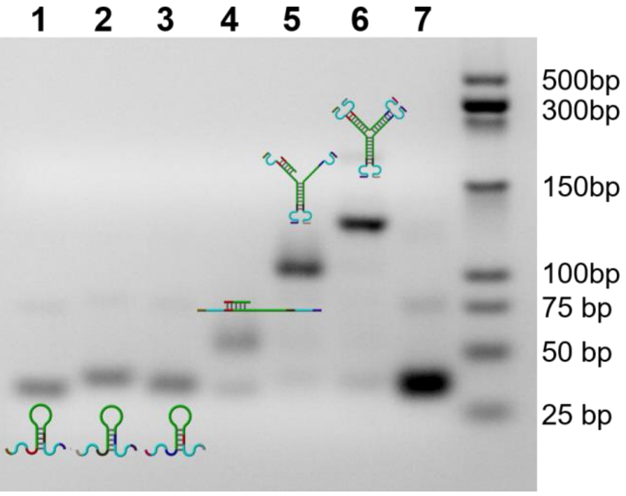

Figure S5. Agarose gel electrophoresis (4%) analysis of the construction of the three-arm DNA junction by branched CHA reaction described in Figure 3. Lane 1: H_{D2}, lane 2: H_{E2}, lane 3: H_{F2}, lane 4: H_{D2} + P_2, lane 5: H_{D2} + H_{E2} + P_2, lane 6: H_{D2} + H_{E2} + H_{F2} + P_2, lane 7: H_{D2} + H_{E2} + H_{F2}. Lanes 1 - 6 show the stepwise construction of three-arm DNA junction upon the addition of P_2, while lane 7 reveals that hairpins could not spontaneously assemble into three-arm DNA junction in the absence of P_2.

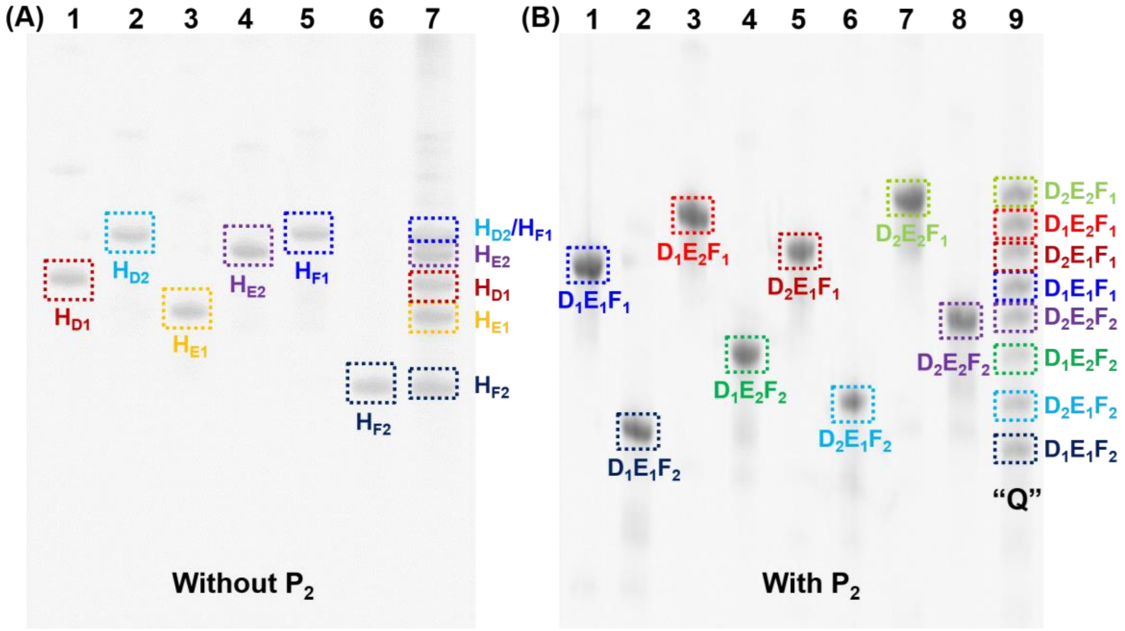

Figure S6. Gel electrophoresis demonstrating primer-P_2-guided evolution of a three-dimensional CDN "Q" shown in Figure 3. (A) Gel electrophoretic image (15% PAGE) of the set of six hairpins in the absence of P_2. Lane 1: H_{D1}, lane 2: H_{D2}, lane 3: H_{E1}, lane 4: H_{E2}, lane 5: H_{F1}, lane 6: H_{F2}, lane 7: the mixture of six hairpins in the absence of P_2. (B) Electrophoretic separated bands (7.5% PAGE) of the CDN "Q", generated upon the P_2-induced branched CHA reaction. Lanes 1-8 correspond to the intact separated constituents: lane 1: $D_1E_1F_1$, lane 2: $D_1E_1F_2$, lane 3: $D_1E_2F_1$, lane 4: $D_1E_2F_2$, lane 5: $D_2E_1F_1$, lane 6: $D_2E_1F_2$, lane 7: $D_2E_2F_1$, lane 8: $D_2E_2F_2$. Lane 9: the separated bands of CDN "Q", generated upon the treatment of six hairpins, H_{D1}-H_{F2} with P_2. The results shown in lane 9 demonstrate the P_2-guided emergence of the CDN "Q" comprising of the eight constituents $D_1E_1F_1$, $D_1E_1F_2$, $D_1E_2F_1$, $D_1E_2F_2$, $D_2E_1F_1$, $D_2E_1F_2$, $D_2E_2F_1$, and $D_2E_2F_2$.

Using ImageJ software and comparing the intensities of the separated bands to those of the individual constituents at known concentrations (1 μM), the contents of the constituents in CDN "Q" generated upon the P_2-induced branched CHA reaction were evaluated, and the results were summarized in Table S3.

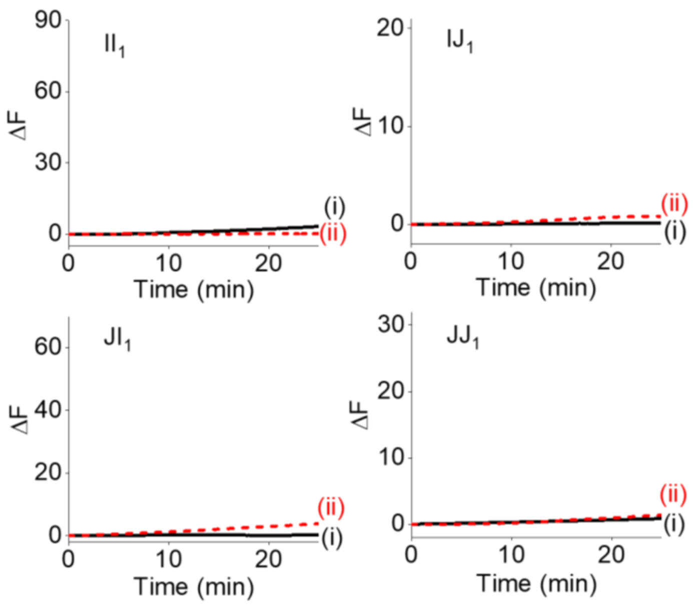

Figure S7. Time-dependent fluorescence changes generated by the Mg^{2+}-ion-dependent DNAzyme reporter units: (i) in the absence of primer P_3, and (ii) upon subjecting P_3 to the separate second-layer CHA module.

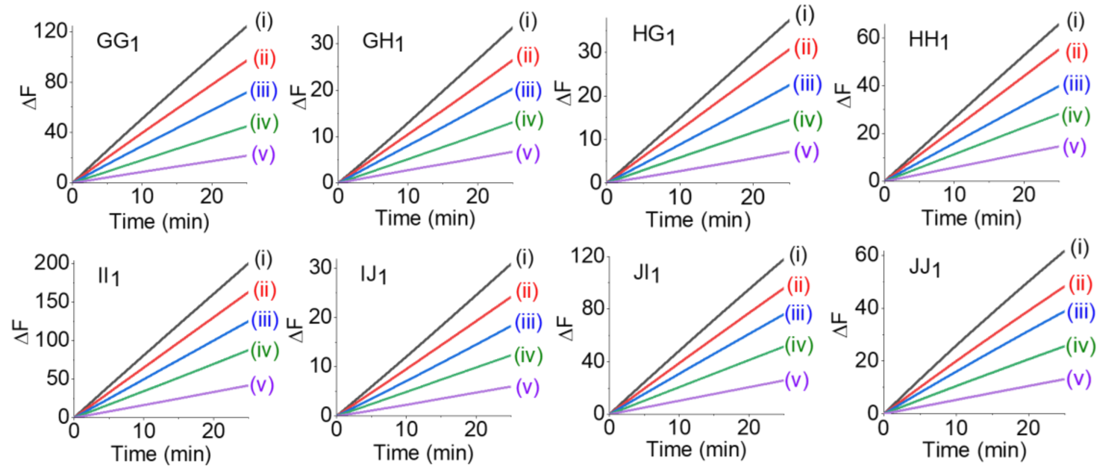

Figure S8. Time-dependent fluorescence changes generated upon the cleavage of the fluorophore/quencher-modified substrates by the respective Mg^{2+}-ion-DNAzyme reporter units associated with the individual intact constituents at variable concentrations: (i) 1 μM, (ii) 0.8 μM, (iii) 0.6 μM, (iv) 0.4 μM, and (v) 0.2 μM.

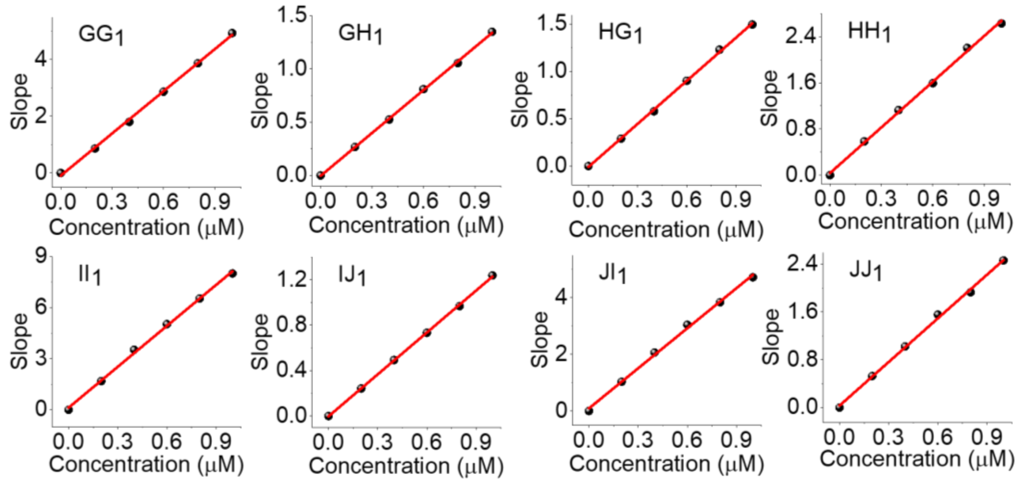

Figure S9. Corresponding calibration curves of the catalytic rates of the different constituents as a function of their concentrations, derived from the data shown in **Figure S8**.

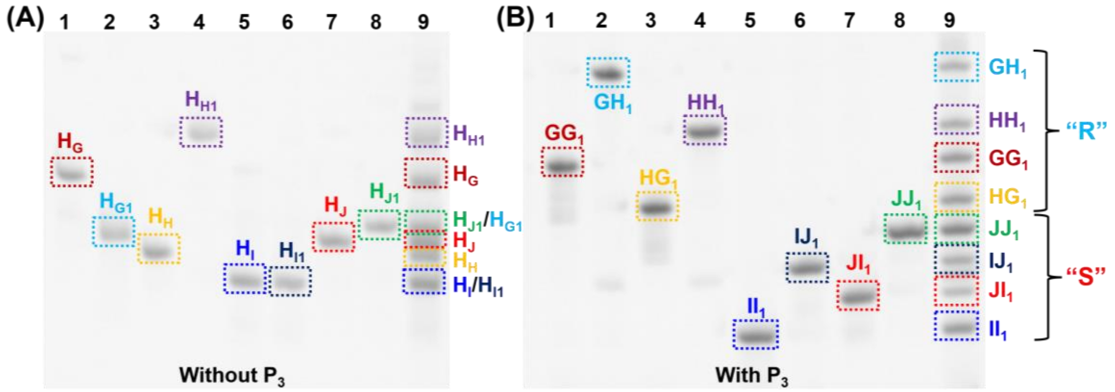

Figure S10. Gel electrophoresis demonstrating the primer-P_3-guided cascaded evolution of CDN "R" and CDN "S", displayed in Figure 4. (A) Electrophoretic bands (15% PAGE) corresponding to the set of eight hairpins in the absence of P_3. Lane 1: H_G, lane 2: H_{G1}, lane 3: H_H, lane 4: H_{H1}, lane 5: H_I, lane 6: H_{I1}, lane 7: H_J, lane 8: H_{J1}, lane 9: the mixture of eight hairpins in the absence of P_3. (B) Electrophoretic separated bands (15% PAGE) corresponding to the CDN "R" and "S", generated upon the P_3-induced two-layer CHA cascade. Lanes 1-8 corresponding to the intact separated constituents: lane 1: GG_1, lane 2: GH_1, lane 3: HG_1, lane 4: HH_1, lane 5: II_1, lane 6: IJ_1, lane 7: JI_1, and lane 8: JJ_1. Lane 9: the separated bands of evolved CDNs "R" and "S", generated upon the treatment of eight hairpins, H_G-H_{J1} with P_3. The results shown in lane 9 demonstrate the P_3-guided cascaded emergence of the CDNs "R" and "S" comprising of the eight constituents GG_1, GH_1, HG_1, HH_1, II_1, IJ_1, JI_1, and JJ_1.

Using ImageJ software and comparing the intensities of the separated bands to those of the individual constituents at known concentrations (1 μM), the contents of the constituents in CDNs "R" and "S" generated upon the P_3-induced two-layer CHA cascade were evaluated, and the results were summarized in Table S4.

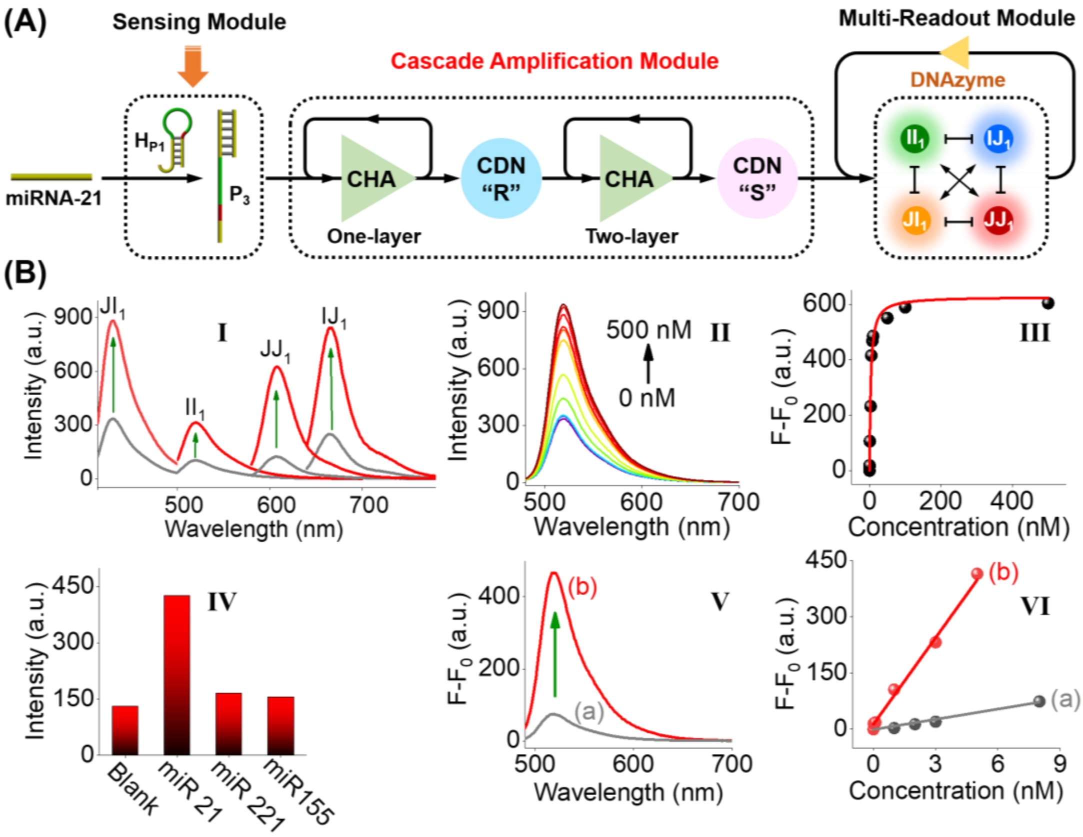

Figure S11. (A) Schematic design of a cascade amplification circuit for detection of miRNA-21 using four fluorescence readout signals generated by DNAzyme reporter units associated with four constituents in CDN "S". (B) Panel I: Multiple fluorescence readout signals for analysis of miRNA-21, generated upon the cleavage of four different fluorophore-/quencher-modified substrates by the respective DNAzyme reporter units associated with four constituents II_1, IJ_1, JI_1, and JJ_1 in evolved CDN "S". Panel II: The fluorescence spectra (using the DNAzyme associated with constituent JI_1 as the readout signal) as a function of variable concentrations of miRNA-21. Panel III: Derived calibration curve corresponding to the fluorescence changes at different concentrations of miRNA-21. Panel IV: Selectivity of miRNA-21 sensing in the form of a bar presentation. Panel V: Fluorescence spectra generated by single-layer CHA (a) and two-layer cascade CHA circuit (b), in the presence of mi-RNA-21, 50 nM. Panel VI: Calibration curves of single-layer CHA (a) and two-layer cascade CHA circuit (b) as a function of variable concentrations of miRNA-21.

The concept of nonenzymatic two-layer CHA cascade amplification circuit and four fluorescence readout signals generated by DNAzyme reporter units associated with constituents in evolved CDN "R" were used to develop a universal sensing platform, as displayed in Figure S11. The miRNA-21 used in the present study is a unique oncogenic biomarker that is overexpressed in most types of cancer. The auxiliary hairpin "H_{P1}" was introduced to recognize and hybridize with miRNA-21 analytes to

release the trigger sequence P_3, and upon opening, it activates the well-established two-layer CHA cascade amplification circuit. Figure S11 schematically depicts two-layer CHA cascade circuit for amplified analysis of miRNA-21. The analyte miRNA-21 hybridizes with and opens the auxiliary hairpin "H_{P1}" to release the trigger sequence P_3. The miRNA-21-triggered release of P_3 stimulates the first-layer CHA to form four duplex constituents GG_1, GH_1, HG_1, and HH_1 comprising CDN "R", which in turn catalyzes the second-layer CHA reaction for the formation of CDN "S" composed of II_1, IJ_1, JI_1, and JJ_1. Each of the constituents in evolved CDN "S" includes a different Mg^{2+}-ion-dependent DNAzyme that catalyzes the cleavage of the respective fluorophore/quencher-modified substrate, thus leading to a substantial fluorescence increase in the DNAzyme amplification stage. That is, the four intercommunicating constituents in the evolved CDN "S" are anticipated to yield four different fluorescence signals, providing four synergistic readout signals for reliable quantitative analysis of miRNA-21. Figure S11B, panel I shows fluorescence intensities generated by DNAzymes associated with four constituents, II_1, IJ_1, JI_1, and JJ_1 in CDN "S" before and after addition of miRNA-21. Upon the addition of miRNA-21, the fluorescence intensities associated with four constituents II_1, IJ_1, JI_1, and JJ_1 are intensified, demonstrating the multiple readout fluorescence signals for sensing of miRNA-21 is, indeed feasible. The sensing performance of the proposed two-layer CHA circuit was investigated using fluorescence intensity associated with constituent JI_1 as the readout signal. Figure S11B, panel II and III, depicts the fluorescence spectra and derived calibration curve in the presence of variable concentrations of miRNA-21, respectively. The detection limit for sensing miRNA-21 corresponds to 10 pM. Figure S11B, panel III, shows the selectivity features corresponding to the analysis of miRNA-21 by two-layer CHA cascade circuit. At a concentration of 50 nM of miRNAs, the signal transduced by two-layer CHA cascade circuit is approximately threefold enhanced in the presence of the target miRNA-21, as compared to the set of foreign miRNAs. To demonstrate the advantage of two-layer cascade circuit, the performance of single-layer CHA was also characterized. Under the same concentration of miRNA-21, the two-

layer cascade system shows a much higher fluorescence intensity than that of single-layer CHA, Figure S11B, panel V, indicating a dramatic signal amplification efficiency of the two-layer CHA cascade. Two-layer CHA cascade amplification circuit reveals sensitivity improvements of two orders of magnitude over single-layer CHA, Figure S11B, panel VI.

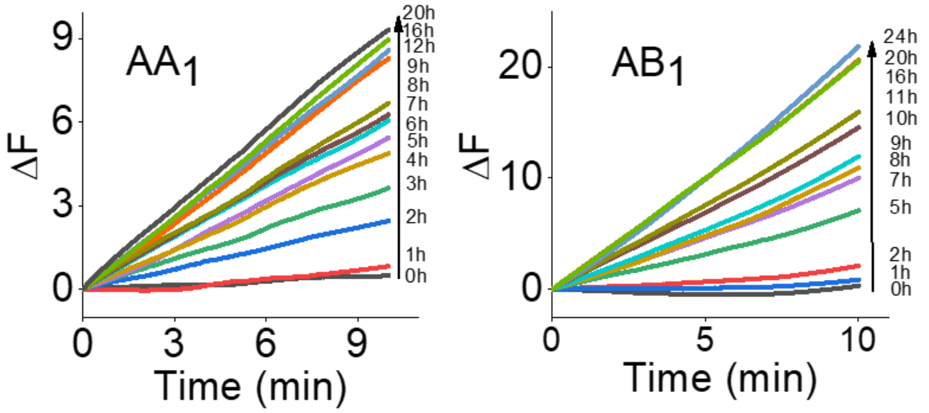

Figure S12. Time-dependent fluorescence changes of constituents AA_1 and AB_1 associated with CDN "O" upon subjecting cross-catalytic feedback circuit to P_1 at different time-interval.

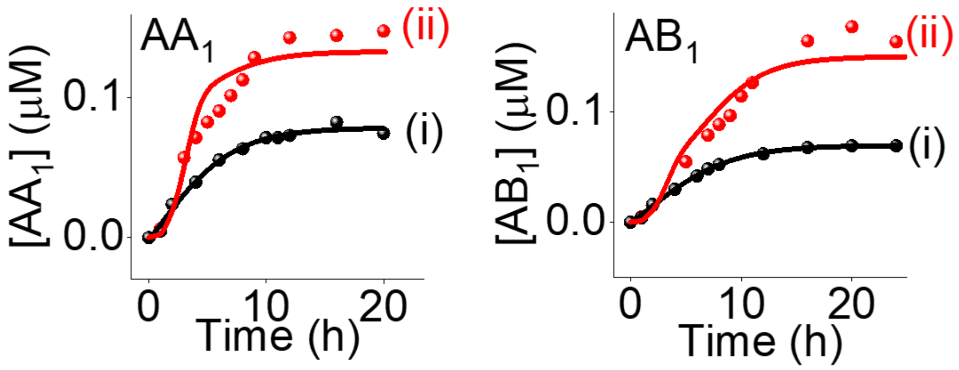

Figure S13. Kinetic analysis of the feedback-driven CHA circuit shown in Figure 5.

As stated in the conclusion section of the paper, the construction of the kinetic model corresponding to the different dynamic CHA reaction frameworks presented in the study and the computational simulation of the experimented results according to the respective kinetic models is an important issue to further characterize the systems. Detailed kinetic models comprising the reaction pathway of the circuits have the potential to predict the behaviour of the systems under variable auxiliary conditions such as the effect of hairpin concentrations involved in the CHA circuits, or the concentrations of the primer on the kinetic behaviours of the systems. While this issue will be addressed in a comprehensive future report, we selected the feedback circuit introduced in Figure 5 as an example to assemble the kinetic model and to simulate computationally the experimental results.

The set of reactions summarized in eq. 1 - eq. 11 account for the temporal feedback driven emergence of the CDN “O” constituents from the set of hairpins H_A, H_{A1}, H_B, and H_{B1}, triggered upon the P_1-triggered activation of the hairpins. Figure 5C, dotted curves represent the experimentally-evaluated dynamic evolution of constituent AA_1 and AB_1. These experimental results were fitted to the kinetic model outlined in eq. 1 - eq. 11. The best fitted computational temporal emergence of AA_1 and of AB_1 are overlayed as solid curves over the experimental data Figure S13. The computationally-simulated rate constants corresponding to the stepwise reaction framework comprising the kinetic model are summarized in Table S5. This framework of rate-constants, may, then, be applied to predict the non-linear temporal emergence of the constituents AA_1 and AB_1 at variable auxiliary conditions of the hairpins/P_1 as inputs.

Kinetic equations of the feedback-driven CHA circuit shown in Figure 5:

$$(1) A + P \underset{k_{-1}}{\overset{k_1}{\rightleftharpoons}} AP$$

$$(2) B + P \underset{k_{-2}}{\overset{k_2}{\rightleftharpoons}} BP$$

$$(3) AP + A1 \underset{k_{-3}}{\overset{k_3}{\rightleftharpoons}} AA1 + P$$

$$(4) AP + B1 \underset{k_{-4}}{\overset{k_4}{\rightleftharpoons}} AB1 + P$$

$$(5) BP + A1 \underset{k_{-5}}{\overset{k_5}{\rightleftharpoons}} BA1 + P$$

$$(6) BP + B1 \underset{k_{-6}}{\overset{k_6}{\rightleftharpoons}} BB1 + P$$

$$(7) AA1 + BB1 \underset{k_{-7}}{\overset{k_7}{\rightleftharpoons}} AB1 + BA1$$

$$(8) AB1 + L1S \underset{k_{-8}}{\overset{k_8}{\rightleftharpoons}} AB1L1S$$

$$(9) AB1L1S \xrightarrow{k_9} AB1 + L1'S + P$$

$$(10) BB1 + L2S \underset{k_{-10}}{\overset{k_{10}}{\rightleftharpoons}} BB1L2S$$

$$(11) BB1L2S \xrightarrow{k_{11}} BB1 + L2'S + P$$

Derivatives:

$$\frac{dA}{dt} = K_{-1}[AP] - K_1[A][P]$$

$$\frac{dP}{dt} = K_{-1}[AP] - K_1[A][P] + K_{-2}[BP] - K_2[B][P] + K_3[AP][A1] - K_{-3}[AA1][P] + K_4[AP][B1] - K_{-4}[AB1][P] +$$
$$K_5[BP][A1] - K_{-5}[BA1][P] + K_6[BP][B1] - K_{-6}[BB1][P] + K_9[AB1L1S] + K_{11}[BB1L2S]$$

$$\frac{dAP}{dt} = K_1[A][P] - K_{-1}[AP] + K_{-3}[AA1][P] - K_3[AP][A1] + K_{-4}[AB1][P] - K_4[AP][B1]$$

$$\frac{dB}{dt} = K_{-2}[BP] - K_2[B][P]$$

$$\frac{dBP}{dt} = K_2[B][P] - K_{-2}[BP] + K_{-5}[BA1][P] - K_5[BP][A1]$$

$$\frac{dA1}{dt} = K_{-3}[AA1][P] - K_3[AP][A1] + K_{-5}[BA1][P] - K_5[BP][A1]$$

$$\frac{dAA1}{dt} = K_3[AP][A1] - K_{-3}[AA1][P] + K_{-7}[AB1][BA1] - K_7[AA1][BB1]$$

$$\frac{dB1}{dt} = K_{-4}[AB1][P] - K_4[AP][B1] + K_{-5}[BA1][P] - K_5[BP][A1]$$

$$\frac{dAB1}{dt} = K_4[AP][B1] - K_{-4}[AB1][P] + K_7[AA1][BB1] - K_{-7}[AB1][BA1] + K_{-8}[AB1L1S] - K_8[AB1][L1S]$$

$$\frac{dBA1}{dt} = K_5[BP][A1] - K_{-5}[BA1][P] + K_7[AA1][BB1] - K_{-7}[AB1][BA1]$$

$$\frac{dBB1}{dt} = K_6[BP][B1] - K_{-6}[BB1][P] + K_{-7}[AB1][BA1] - K_7[AA1][BB1] + K_{-10}[BB1L2S] - K_{10}[BB1][L2S] + K_{11}[BB1L2S]$$

$$\frac{dL1S}{dt} = K_{-8}[AB1L1S] - K_8[AB1][L1S]$$

$$\frac{dAB1L1S}{dt} = K_8[AB1][L1S] - K_{-8}[AB1L1S] - K_9[AB1L1S]$$

$$\frac{dL1'S}{dt} = K_9[AB1L1S]$$

$$\frac{dL2S}{dt} = K_{-10}[BB1L2S] - K_{10}[BB1][L2S]$$

$$\frac{dBB1L2}{dt} = K_{10}[BB1][L2S] - K_{-10}[BB1L2S] - K_{11}[BB1L2S]$$

$$\frac{dL2'S}{dt} = K_{11}[BB1L2S]$$

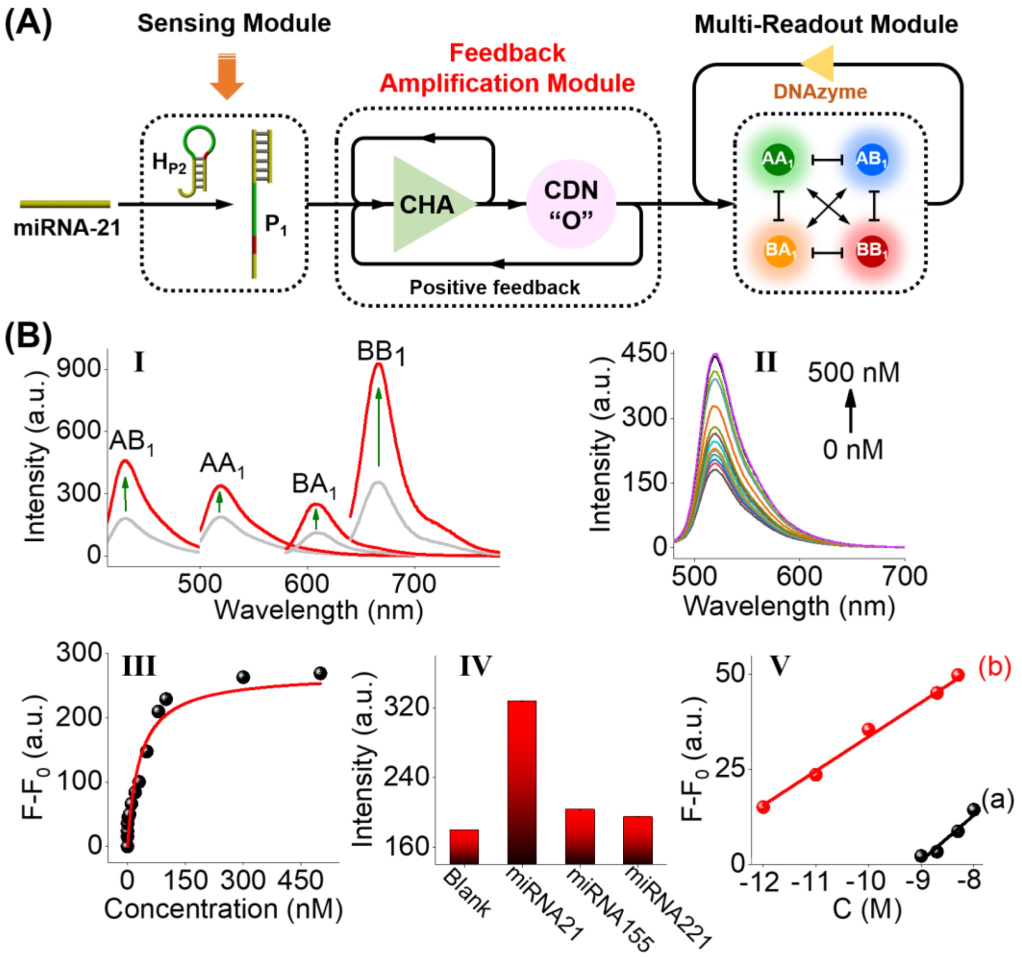

Figure S14. (A) Schematic design of a feedback amplification circuit for detection of miRNA-21 using four fluorescence readout signals generated by DNAzyme reporter units associated with four constituents in CDN "O". (B) Panel I: Multiple fluorescence readout signals for analysis of miRNA-21, generated upon the cleavage of four different fluorophore-/quencher-modified substrates by the respective DNAzyme reporter units associated with four constituents AA_1, AB_1, BA_1, and BB_1 in evolved CDN "O". Panel II: The fluorescence spectra (using the DNAzyme associated with constituent AB_1 as the readout signal) as a function of variable concentrations of miRNA-21. Panel III: Derived calibration curve corresponding to the fluorescence changes at different concentrations of miRNA-21. Panel IV: Selectivity of miRNA-21 sensing in the form of a bar presentation. Panel V: Calibration curves of non-feedback circuit (a) and feedback circuit (b) as a function of variable concentrations of miRNA-21.

Similar to the two-layer cascade circuit, the feedback amplification circuit was also used to develop a universal sensing platform for analysis of miRNA-21, as displayed in Figure S14. The auxiliary hairpin "H_{P2}" was introduced to recognize and hybridize with miRNA-21 analyte to release the trigger sequence P_1, and upon opening, it activates the well-established feedback amplification circuit. Figure S14 schematically

depicts the feedback amplification circuit for analysis of miRNA-21. The analyte miRNA-21 hybridizes with and opens the auxiliary hairpin "H_{P2}" to release the trigger sequence P_1. The miRNA-21-triggered release of P_1 stimulates the CHA to form four duplex constituents AA_1, AB_1, BA_1, and BB_1 comprising CDN "O". Subsequently, the DNAzymes associated with constituents BA_1 and BB_1 cleave the caged substrates S_1 and S_2, respectively, to continuously generate protected P_1 sequences that could, in turn, catalyze the CHA reaction. Each of the constituents in evolved CDN "O" includes a different Mg^{2+}-ion-dependent DNAzyme that catalyzes the cleavage of the respective fluorophore/quencher-modified substrate, thus leading to a substantial fluorescence increase in the DNAzyme amplification stage. That is, the four intercommunicating constituents in the evolved CDN "O" are anticipated to yield four different fluorescence signals, providing four synergistic readout signals for reliable quantitative analysis of miRNA-21. Figure S14B, panel I shows fluorescence intensities generated by DNAzymes associated with four constituents, AA_1, AB_1, BA_1, and BB_1 in CDN "O" before and after addition of miRNA-21. Upon the addition of miRNA-21, the fluorescence intensities associated with four constituents AA_1, AB_1, BA_1, and BB_1 are intensified, demonstrating the multiple readout fluorescence signals for sensing of miRNA-21 is, indeed feasible. The sensing performance of the proposed feedback amplification circuit was investigated using fluorescence intensity associated with constituent AB_1 as the readout signal. Figure S14B, panel II and III, depicts the fluorescence spectra and derived calibration curve in the presence of variable concentrations of miRNA-21, respectively. The detection limit for sensing miRNA-21 corresponds to 1 pM. Figure S14B, panel IV, shows the selectivity features corresponding to the analysis of miRNA-21 by feedback amplification circuit. At a concentration of 50 nM of miRNAs, the signal transduced by feedback circuit is approximately twofold enhanced in the presence of the target miRNA-21, as compared to the set of foreign miRNAs. To demonstrate the advantage of the feedback amplification circuit, the performance of non-feedback circuit was also characterized. Under the same concentration of miRNA-21, the feedback system shows a much higher

fluorescence intensity than that of non-feedback circuit, Figure S14B, panel V, indicating a dramatic signal amplification efficiency of the feedback circuit. The feedback amplification circuit reveals sensitivity improvements of three orders of magnitude over non-feedback circuit, Figure S14B, panel V.

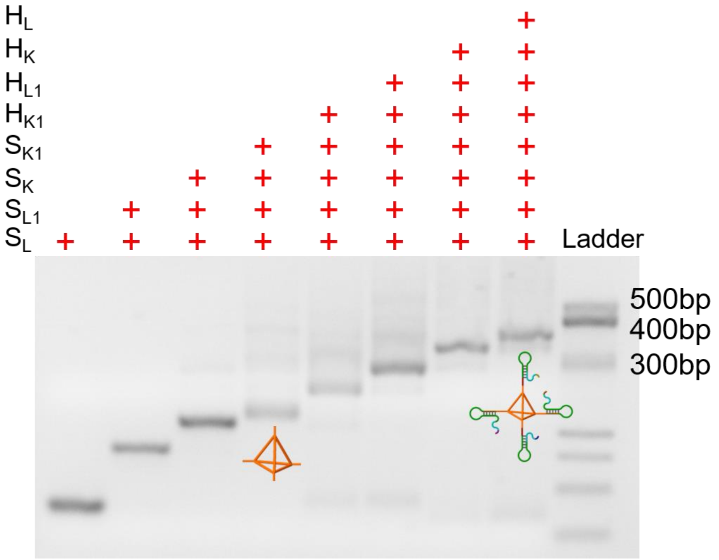

Figure S15. Agarose gel (4%) electrophoretic image demonstrating the formation of hairpins-functionalized tetrahedra.

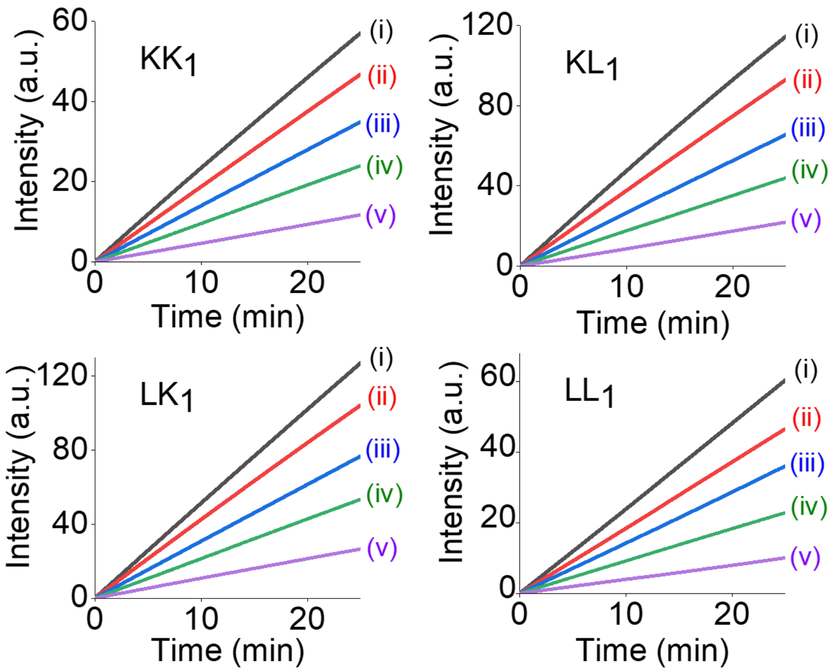

Figure S16. Time-dependent fluorescence changes generated upon the cleavage of the fluorophore/quencher-modified substrates by the respective Mg^{2+}-ion-DNAzyme reporter units associated with the individual intact constituents at variable concentrations: (i) 1 µM, (ii) 0.8 µM, (iii) 0.6 µM, (iv) 0.4 µM, and (v) 0.2 µM.

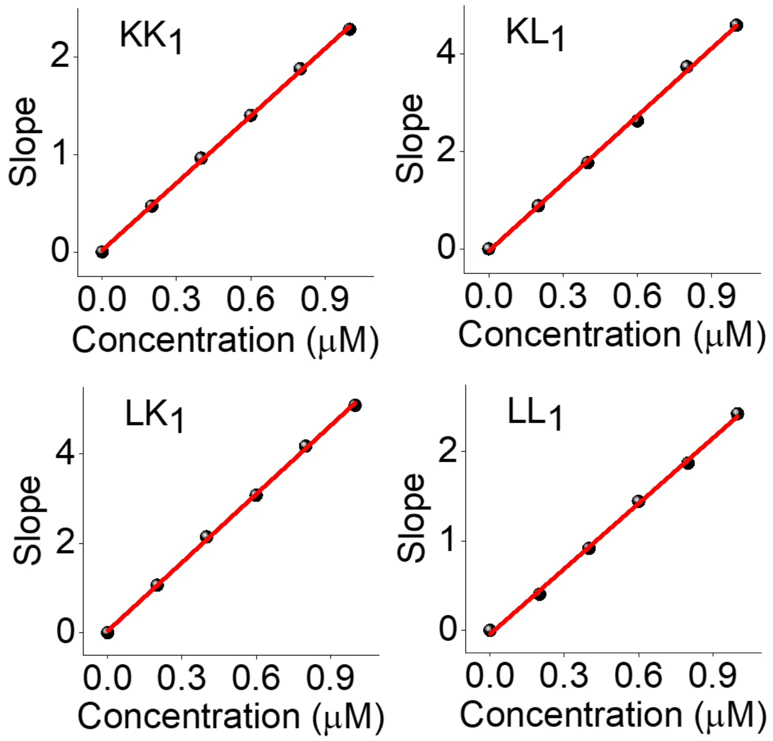

Figure S17. Corresponding calibration curves of the catalytic rates of the different constituents as a function of their concentrations, derived from the data shown in **Figure S16**.

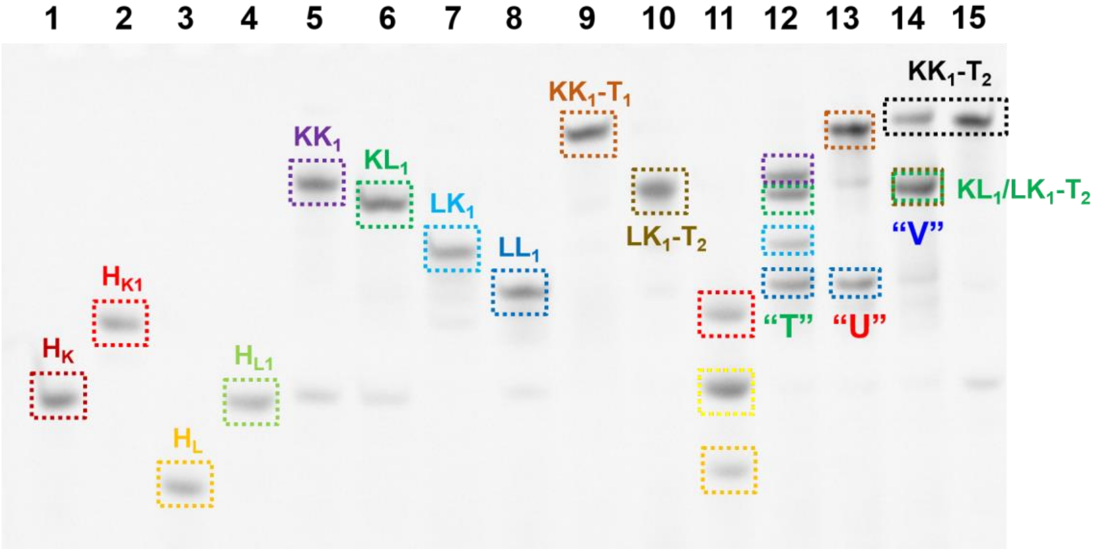

Figure S18. Gel electrophoresis (12% PAGE) demonstrating primer-P_4-guided evolution and orthogonal-triggered transitions of CDN "T" into CDN "U" and CDN "V", displayed in Figure 6. For comparison, the bands of the individual hairpins, namely, H_K (lane 1), H_{K1} (lane 2), H_L (lane 3), and H_{L1} (lane 4), and the separated intact constituents, KK_1 (lane 5), KL_1 (lane 6), LK_1 (lane 7), LL_1 (lane 8), KK_1-T_1 (lane 9), and LK_1-T_2 (lane 10) are provided. Lane 11 corresponds to the mixture of four hairpins, H_K-H_{L1} in the absence of primer P_4. Lane 12 shows the separated constituents of CDN "T", generated upon the P_4-triggered activation of spatially-localized CHA on tetrahedron. Lane 13 shows the separated constituents of CDN "U". Lane 14 shows the separated constituents of CDN "V". Further control reference, intact constituent KK_1-T_2 is presented in Lane 15.

Using ImageJ software and comparing the intensities of the separated bands to those of the individual constituents at known concentrations (1 μM), the contents of the constituents in CDNs "T", "U" and "V" were evaluated, and the results were summarized in Table S6.

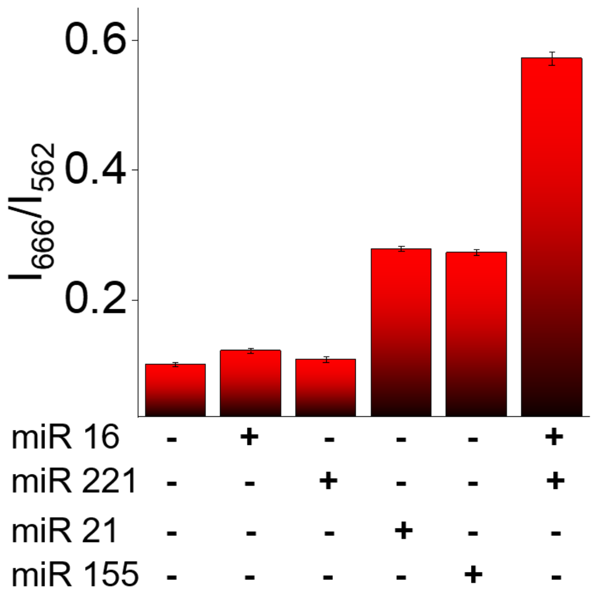

Figure S19. Selectivity of localized CHA sensor in the form of a bar presentation. Error bars derived from N = 3 experiments.

Table S1. The contents of the constituents in CDN "O" evolved by the primer P_1-triggered activation of CHA reaction, shown in Figure 1.

CDN	AA_1	AB_1	BA_1	BB_1
O[a]	0.51	0.51	0.61	0.48

[a] The contents of the constituents (μM) were evaluated from the quantitative electrophoretic experiment in Figure S3.

Table S2. The contents of the constituents in CDN "P" evolved by the primer P_1-triggered activation of CHA reaction shown in Figure 2.

**CDNs**	**AA_1**	**AB_1**	**AC_1**	**BA_1**	**BB_1**	**BC_1**	**CA_1**	**CB_1**	**CC_1**
**P**[a]	0.35	0.30	0.18	0.41	0.41	0.16	0.22	0.18	0.48
**P**[b]	0.38	0.32	0.33	0.37	0.42	0.28	0.39	0.28	0.41

[a] The contents of the constituents (μM) were evaluated from the time-dependent fluorescence changes generated by the reporter units and using appropriate calibration curve in Figures S1 and S2.

[b] The contents of the constituents (μM) were evaluated from the quantitative electrophoretic experiment in Figure S4.

Table S3. The composition of the constituents in CDN "Q" evolved by the primer P_2-triggered activation of the branched CHA, shown in Figure 3.

**CDN**	**$D_1E_1F_1$**	**$D_1E_1F_2$**	**$D_1E_2F_1$**	**$D_1E_2F_2$**	**$D_2E_1F_1$**	**$D_2E_1F_2$**	**$D_2E_2F_1$**	**$D_2E_2F_2$**
**Q**[a]	0.26	0.31	0.27	0.19	0.25	0.25	0.28	0.21

[a] The contents of the constituents (μM) were evaluated from the quantitative electrophoretic experiment in Figure S6.

It should be noted that the concentrations of the constituents are not equal. This is consistent with the fact that the concentrations of the constituents are dictated by the relative stabilities of the constituents that might include secondary intra-constituent structure, and eventually further interactions with auxiliary strands.

Table S4. The composition of the constituents in CDN "R" and CDN "S" evolved by the primer P_3-triggered activation of the two-layer CHA cascade circuit, shown in Figure 4.

**CDN**	**GG_1**	**GH_1**	**HG_1**	**HH_1**	**CDN**	**II_1**	**IJ_1**	**JI_1**	**JJ_1**
**R**[a]	0.53	0.57	0.51	0.47	**S**[a]	0.41	0.34	0.49	0.53
**R**[b]	0.55	0.49	0.58	0.41	**S**[b]	0.52	0.43	0.41	0.58

[a] The contents of the constituents (μM) were evaluated from the time-dependent fluorescence changes generated by the reporter units and using appropriate calibration curve in Figures S8 and S9.

[b] The contents of the constituent (μM) were evaluated from the quantitative electrophoretic experiments in Figure S10.

Table S5. Rate constants derived from the feedback-driven CHA circuit shown in Figure 5.

k_1	1.32 μm^{-1} min^{-1}	k_7	1.02 μm^{-1} min^{-1}
k_{-1}	0.12 min^{-1}	k_{-7}	1.36 μm^{-2} min^{-1}
k_2	10.2 μm^{-1} min^{-1}	k_8	0.96 μm^{-1} min^{-1}
k_{-2}	0.01 min^{-1}	k_{-8}	0.25 min^{-1}
k_3	1.06 μm^{-1} min^{-1}	k_9	0.01 min^{-1}
k_{-3}	0.54 μm^{-1} min^{-1}	k_{10}	0.86 μm^{-1} min^{-1}
k_4	1.02 μm^{-1} min^{-1}	k_{-10}	0.31 min^{-1}
k_{-4}	0.32 μm^{-1} min^{-1}	k_{11}	0.01 min^{-1}
k_5	1.18 μm^{-1} min^{-1}		
k_{-5}	0.91 μm^{-1} min^{-1}		
k_6	1.02 μm^{-1} min^{-1}		
k_{-6}	0.85 μm^{-1} min^{-1}		

Table S6. The composition of the constituents in CDN "T", CDN "U" and CDN "V", shown in Figure 6.

**CDNs**	**KK_1**	**KL_1**	**LK_1**	**LL_1**
T[a]	0.57	0.51	0.62	0.43
T[b]	0.59	0.54	0.52	0.40
U[a]	0.88	0.18	0.19	0.80
U[b]	0.90	0.22	0	0.72
V[a]	0.23	0.78	1.00	0.17
V[b]	0.25	-	-	0.20

[a] The contents of the constituents (μM) were evaluated from the time-dependent fluorescence changes generated by the reporter units and using appropriate calibration curve in Figures S16 and S17.

[b] The contents of the constituents (μM) were evaluated from the quantitative electrophoretic experiment in Figure S18.
